# Negative Regulators of Insulin Signaling Revealed in a Genome-Wide Functional Screen

**DOI:** 10.1371/journal.pone.0006871

**Published:** 2009-09-03

**Authors:** Shih-Min A. Huang, Michael K. Hancock, Jeffrey L. Pitman, Anthony P. Orth, Nicholas Gekakis

**Affiliations:** 1 Genomics Institute of the Novartis Research Foundation, San Diego, California, United States of America; 2 The Scripps Research Institute, La Jolla, California, United States of America; University of Bremen, Germany

## Abstract

**Background:**

Type 2 diabetes develops due to a combination of insulin resistance and β-cell failure and current therapeutics aim at both of these underlying causes. Several negative regulators of insulin signaling are known and are the subject of drug discovery efforts. We sought to identify novel contributors to insulin resistance and hence potentially novel targets for therapeutic intervention.

**Methodology:**

An arrayed cDNA library encoding 18,441 human transcripts was screened for inhibitors of insulin signaling and revealed known inhibitors and numerous potential novel regulators. The novel hits included proteins of various functional classes such as kinases, phosphatases, transcription factors, and GTPase associated proteins. A series of secondary assays confirmed the relevance of the primary screen hits to insulin signaling and provided further insight into their modes of action.

**Conclusion/Significance:**

Among the novel hits was PALD (KIAA1274, paladin), a previously uncharacterized protein that when overexpressed led to inhibition of insulin's ability to down regulate a FOXO1A-driven reporter gene, reduced upstream insulin-stimulated AKT phosphorylation, and decreased insulin receptor (IR) abundance. Conversely, knockdown of PALD gene expression resulted in increased IR abundance, enhanced insulin-stimulated AKT phosphorylation, and an improvement in insulin's ability to suppress FOXO1A-driven reporter gene activity. The present data demonstrate that the application of arrayed genome-wide screening technologies to insulin signaling is fruitful and is likely to reveal novel drug targets for insulin resistance and the metabolic syndrome.

## Introduction

Approximately 143 million people worldwide are afflicted by type 2 diabetes (T2D). Clinical and experimental data have demonstrated that T2D is highly correlated with the induction of insulin resistance. Finding negative modulators of insulin signaling is therefore of enormous scientific and therapeutic importance. Insulin activates two major signaling pathways, namely the phosphatidylinositol-3-OH kinase (PI(3)K)-AKT and RAS-MAPK pathways [1]. While the RAS-MAPK pathway regulates cell growth, PI(3)K-AKT signaling is thought to be the key pathway by which insulin controls metabolic processes. Several insulin signaling inhibitors (e.g. PTP1B, PTEN, and IKKβ) have already been found [2]–[5]. Given the complexity of insulin signaling, many more are likely to be discovered. For example, TRB3, a CDC25 binding protein homolog, has recently been reported to down-regulate hepatic AKT activation by insulin [6].

In the liver, insulin down regulates glucose production in part by repressing the transcription of the glucose-6-phosphatase (G6Pase) gene via a well-documented PI(3)K-AKT-FOXO1A phosphorylation cascade [7]. Taking advantage of recent advances in functional profiling technology, we initiated a cDNA screen using the promoter of the G6Pase catalytic subunit driving luciferase expression as an insulin responsive reporter (G6Pase-Luc), with the objective of finding negative modulators of insulin signaling. In this report we describe the identification and initial functional characterization of novel cDNA inhibitors of insulin signaling, especially the potential regulatory role of the previously uncharacterized protein PALD on IR signaling.

## Results

**Figure 1:**
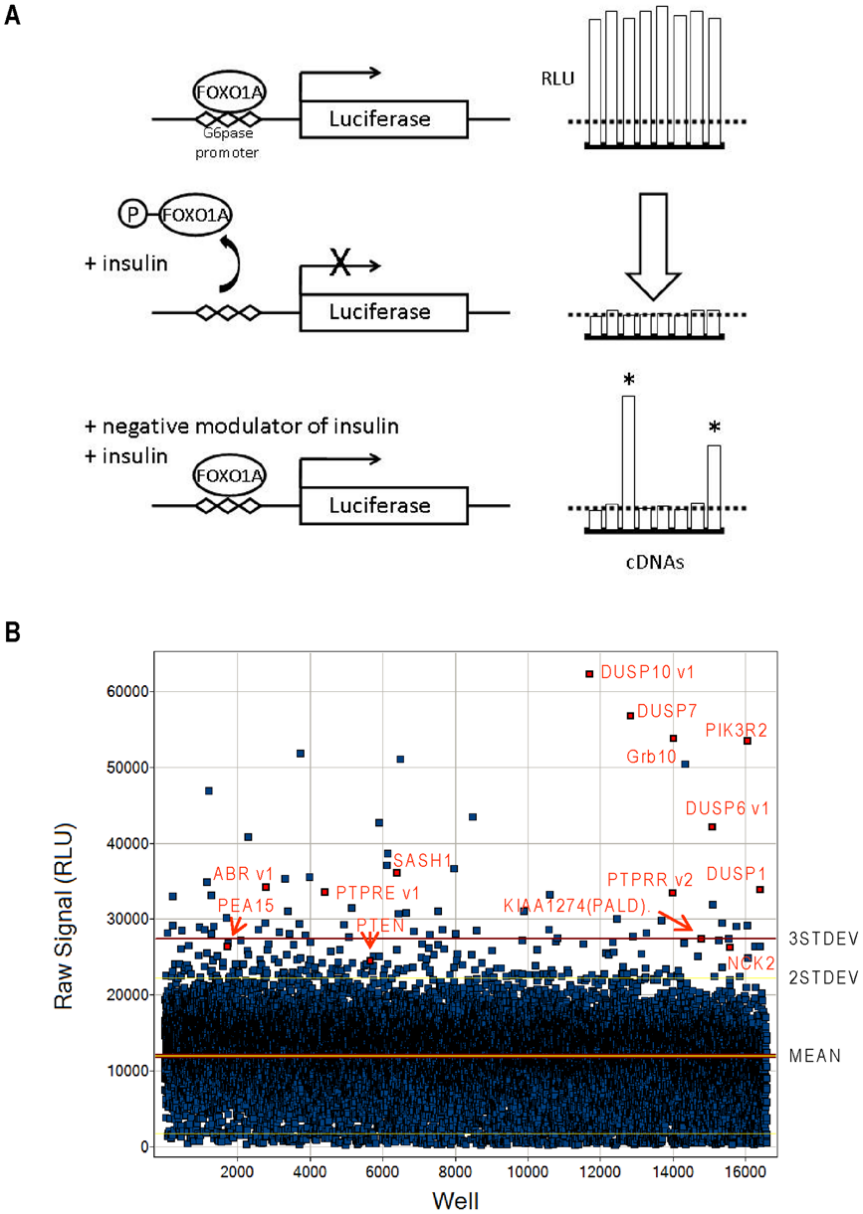
Genome-wide screen for negative modulators of insulin signaling. (A) Scheme illustrating screen design. The G6Pase-Luc reporter is activated by FOXO1A transcription factor and cAMP treatment in the absence of insulin. Upon treatment of cells with insulin, a phosphorylation cascade is induced that leads to inactivation of FOXO1A and repression of G6Pase-Luc activity. In the presence of a cDNA-encoded inhibitor of insulin signaling however, G6Pase-Luc activity is restored. (B) Single-well luminescent signals of the 16,581 high-quality cDNA data set obtained from screening an arrayed library encoding 18,441 human transcripts. The luminescent signal is plotted on the y-axis as relative light units (RLU) with the corresponding well number on the x-axis. Representative hits are labeled in red and denoted as gene symbol plus transcript variant number, as applicable, and include: PTEN, PIK3R2, GRB10, PEA15, NCK2, ABR v1, SASH1, KIAA1274 (PALD), DUSP1, DUSP6 v1, DUSP7, DUSP10 v1, PTPRE v1, and PTPRR v2.

We hypothesized that forced expression of a negative modulator of insulin signaling would de-repress G6Pase-Luc reporter activity in the presence of insulin (Figure 1A). The screen was optimized in readily transfectable Chinese Hamster Ovary (CHO-K1) cells, which are responsive to insulin stimulation as demonstrated by the induction of AKT phosphorylation (Figure S1A). In addition, CHO-K1 cells express an insignificant amount of FOXO1A transcription factor and G6Pase-Luc is inactive in these cells without exogenous expression of FOXO1A (Figure S1B and C). Subsequently, co-transfection of FOXO1A with a genome-scale collection of 18,441 purported full length human cDNAs was performed, yielding 16,581 usable data points and 161 hits with reporter activities greater than two standard deviations above the mean (Figure 1B).

**Figure 2:**
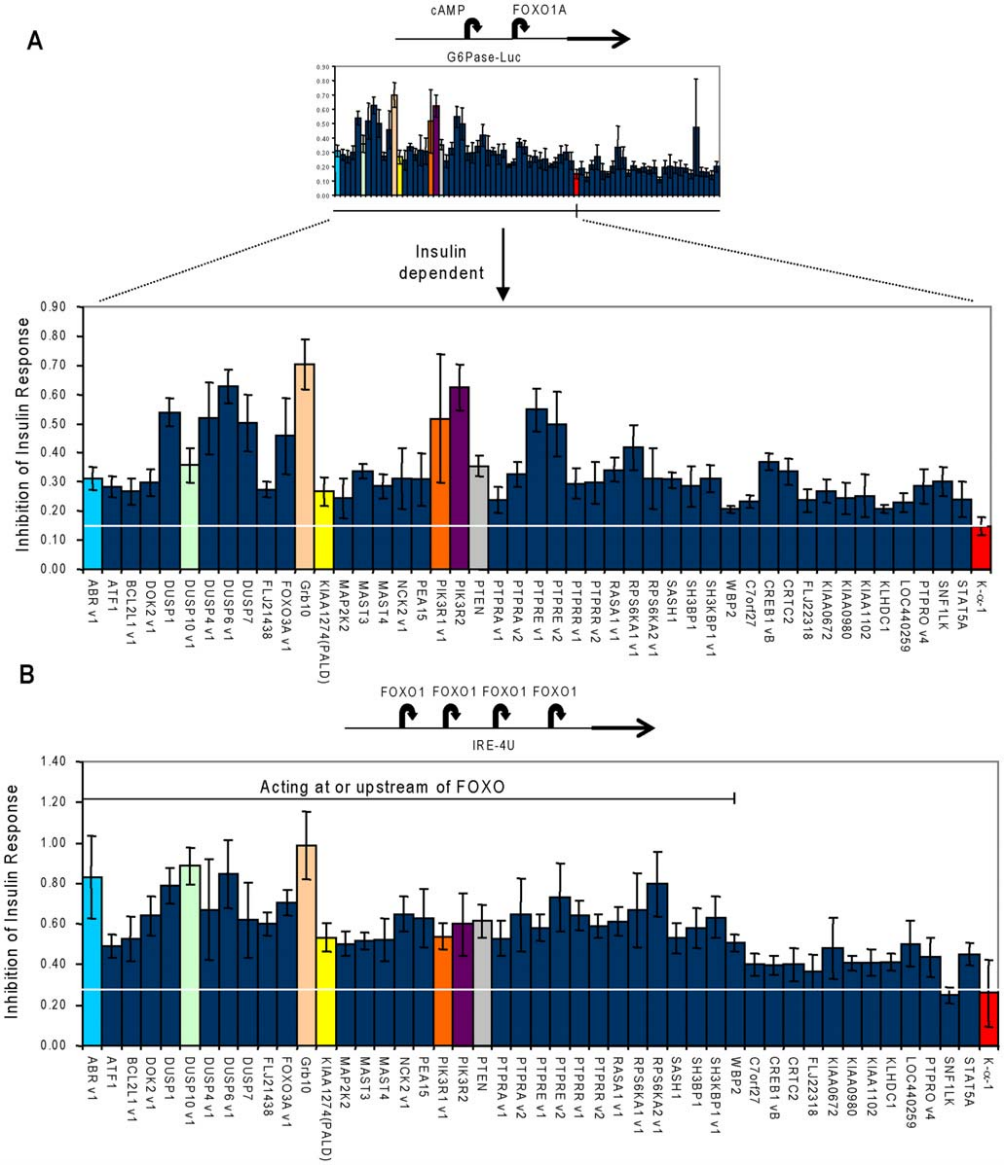
Characterization of hits from the primary screen. (A) Inhibition of insulin's repression of the G6Pase-Luc reporter. Seventy-one selected hits were retested in the primary assay in the presence and absence of insulin. Inhibition of insulin response was defined as the ratio of luciferase activity in the presence of insulin to that in the absence of insulin, which is positively correlated with the extent of inhibition of the insulin signal. The cDNAs were filtered based on their ability to significantly (p<0.05) inhibit insulin signaling in an insulin-dependent manner relative to the negative control, α-tubulin (K-α-1). (B) Inhibition of insulin effect on IRE-4U reporter. The 46 cDNAs demonstrated to inhibit insulin signaling were assayed with a FOXO1A-dependent promoter (IRE-4U). Similar to (A) above, the IRE-4U luciferase activity in the presence of insulin was divided by that in the absence of insulin. This ratio is positively correlated with the extent of inhibition of FOXO1A-dependent transcription by insulin. Hits acting at or upstream of FOXO1A were those that had a ratio greater than that of the negative control, K-α-1 at p<0.05.

We chose 71 primary screen hits for detailed follow-up based on assay activity (i.e., generally favored those hits with highest primary screen activity, although several novel hits with lesser activities still above the two standard deviation cut-off were also included), novelty (i.e., included those hits for which little or no functional information has previously been reported), and predicted functions (i.e., included hits with predicted signal transduction functions such as kinases, phosphatases, and adaptors, while with a few exceptions excluding transcription factor-related hits since these would be predicted to have a higher “nonspecific” hit rate in our screen). First, to determine whether activation of the reporter by the hits was specifically due to inhibition of insulin signaling, we repeated the G6Pase-Luc reporter assay with and without insulin and normalized the reporter activity in the presence of insulin to that in the absence of insulin (Figure 2A). The resulting ratio is directly correlated with the extent of insulin inhibition. For example, whereas overexpression of a negative control α-tubulin (K-α-1) resulted in a minimal ratio of 0.15 (luciferase activity in the presence of insulin is 15% of that in the absence of insulin), expression of PTEN and GRB10, both known inhibitors of insulin signaling, yielded more than two-fold higher inhibition ratios of 0.35 and 0.7 respectively (Figure 2A). Statistically, of the 71 cDNAs examined, 46 significantly overcame insulin's repression of G6Pase promoter activity compared to the negative control, K-α-1 (Figure 2A and Table S1). Importantly, dual specificity phosphatase (DUSP) family members identified in the original screen (DUSP1, 4, 5, 6, 7, and 10) were positive in the insulin-dependency test, while other DUSP family members (DUSP2, 3, & 14) were negative in both the primary and initial secondary screening, indicating specificity among DUSP family members in their effects on insulin signaling and excluding generalized phosphatase overexpression as a source of experimental artifacts.

To obtain initial mechanistic insights we next sought to place secondary hits in the context of two major components of insulin signaling, FOXO1A and AKT. To examine reliance upon FOXO1A, we used a FOXO1A-dependent reporter (IRE-4U) containing four copies of the FOXO1A binding motifs from the G6Pase promoter [8] to analyze the effect of the 46 insulin signaling inhibitors on reporter activity in the presence and absence of insulin. As observed with the G6Pase-Luc reporter assay, PTEN (0.60) and GRB10 (0.99) exhibited at least two-fold greater insulin inhibition ratios than the K-α-1 control (0.26) in the context of the IRE-4U reporter assay. 34 of the 46 insulin-dependent cDNAs also significantly inhibited insulin signaling on the FOXO1A-dependent reporter as compared to K-α-1 (Figure 2B and Table S2) indicating that they act at or upstream of FOXO1A.

**Figure 3:**
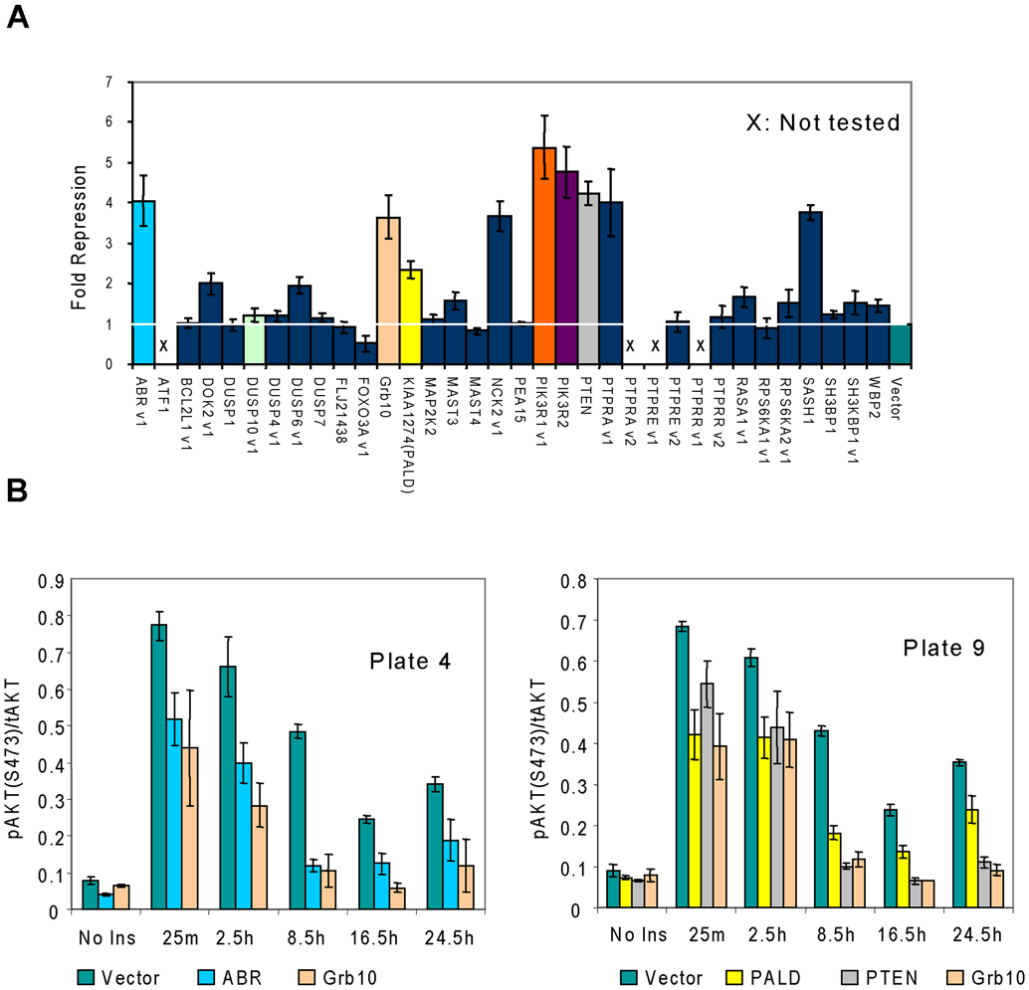
Inhibition of insulin-stimulated Akt phosphorylation by screen hits. (A) Selected screen hits that inhibit insulin signaling at or upstream of FOXO1A were assayed across an insulin treatment time course for their ability to inhibit insulin-induced AKT phosphorylation using a multiplex immunoassay that simultaneously measures phospho-(Ser473) and total AKT levels. Shown here are representative data from a single time point, 8.5 hours post insulin treatment. For the complete time-course data set see Table S3 and Figure S2. (B) Complete time-course data for inhibition of insulin-stimulated AKT phosphorylation by ABR and PALD. For comparison purposes, shown here are the insulin treatment time-course results for two of the novel insulin signaling hits, ABR and PALD, relative to well-known inhibitors, GRB10 and PTEN, and vector control. The individual microplate data shown here are displayed as the pAKT/tAKT ratio on the y-axis with the insulin-treatment time-points denoted on the x-axis. For additional data sets, see Table S3 and Figure S2.

Since insulin regulates FOXO1A transcriptional activity predominately through AKT [9], the effect of overexpression of screen hits on insulin-induced AKT phosphorylation was investigated at six time points over a 24 hour period using a high throughput multiplex immunoassay that detects total AKT and phosphoAKT (S473) proteins simultaneously. Illustrated in Figure 3A are representative data from a single time point 8.5 hours after insulin stimulation (see Table S3 and Figure S2 for the complete data set). For each cDNA, the raw signal of pAKT was first normalized to the raw signal of total AKT (pAKT/total AKT). The fold repression of the insulin response was then calculated by dividing (pAKT/total AKT) for the control (vector only) by (pAKT/total AKT) for a given cDNA. Consistent with previous reports demonstrating their ability to modulate insulin signaling when overexpressed [10]–[12], GRB10, PTEN, PIK3R1, and PIK3R2 inhibited insulin-induced AKT phosphorylation 3.7-, 4.2-, 5.4-, and 4.8-fold respectively. In Figure 3B are representative time course data for two of the novel screen hits, ABR and PALD, displayed as the raw signal of pAKT normalized to the raw signal of total AKT. Overexpression of ABR or PALD clearly inhibited insulin-stimulated AKT phosphorylation at all time points tested (Figure 3B). Based on the data in Figures 3A, 3B, and the complete time course (Supplemental Information, Table S3 and Figure S2), we found that ABR, DOK2, NCK2, PALD, PTPRA, RPS6KA2, SASH1, and WBP2 significantly inhibited insulin-stimulated AKT phosphorylation at all time points compared to the vector control.

**Figure 4:**
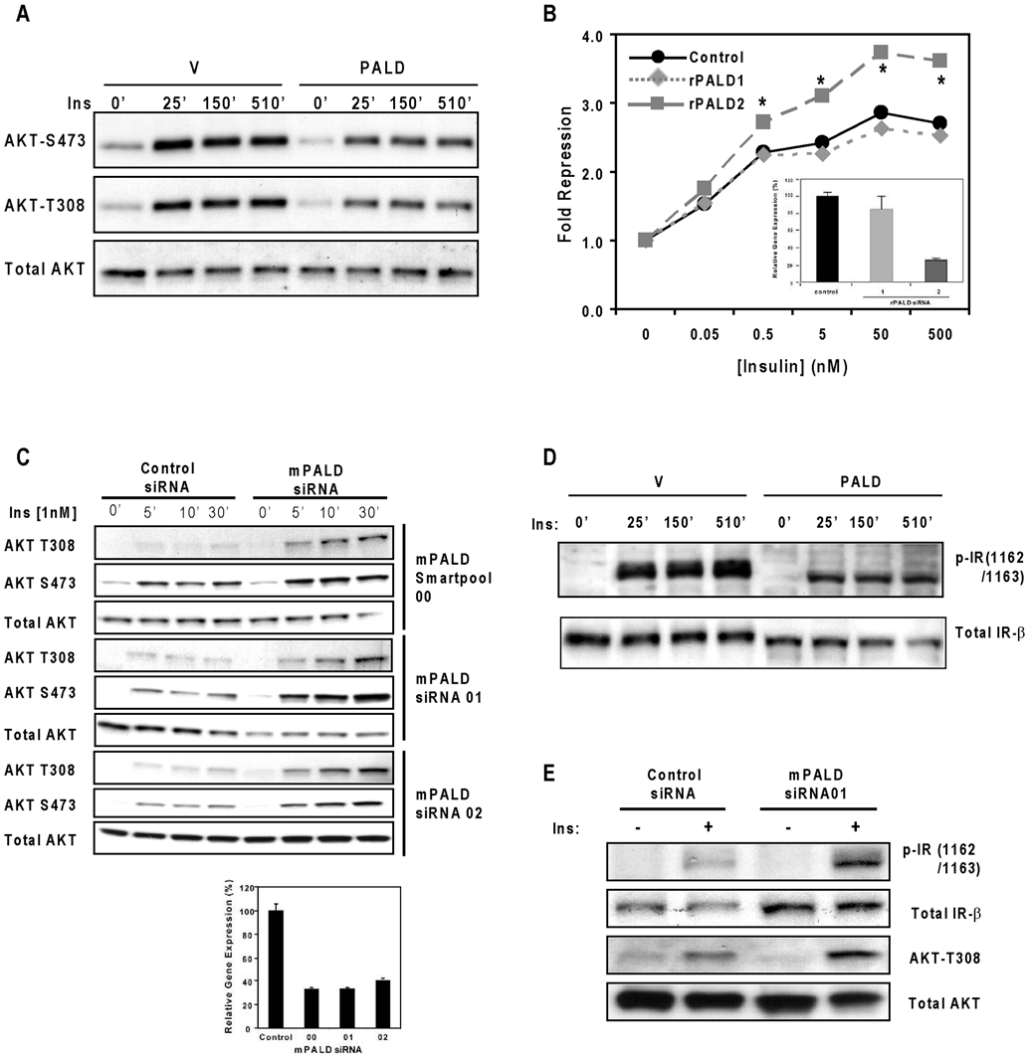
PALD overexpression inhibits insulin–induced AKT phosphorylation while PALD knockdown improves insulin response and enhances insulin-induced AKT phosphorylation. (A) COS7 cells transfected with empty vector or human PALD were serum starved prior to treatment with 50 nM insulin for the times indicated and analyzed for AKT phosphorylation (Ser473 and Thr308) and total AKT expression by Western blotting. (B) H4IIE rat liver cells containing an integrated G6Pase-Luc reporter were electroporated with control siRNA or siRNAs against rat PALD. The extent of PALD mRNA knockdown was measured by qPCR 72 hours after transfection (inset) and inhibition of the luciferase activity in serum-starved cells by insulin (fold repression) was determined. *P<0.01, compared with control siRNA. (C) mPALD knockdown enhances insulin-induced AKT phosphorylation. C2C12 mouse myoblast cells were transfected with control siRNA, pooled mPALD siRNA (mPALD siRNA00), or the individual mPALD siRNAs (mPALD siRNA01 and mPALD siRNA02), serum starved overnight, and treated with insulin for the indicated times before PALD mRNA was measured by qPCR (lower panel) and AKT phosphorylation was analyzed by Western blotting (upper panel). (D) The COS7 cells transfected in (A) were also analyzed for insulin receptor (IR) phosphorylation and IR β subunit expression by Western blotting. (E) mPALD knockdown enhances insulin-stimulated IR phosphorylation and increases IR abundance. C2C12 cells transfected with control siRNA or mPALD siRNA01 were serum starved overnight, treated with 1 nM insulin, and analyzed for phospho IR and total IR by Western blotting. Phospho AKT was included as a control for insulin action and total AKT was included as a loading control.

Among the novel hits shown to affect AKT phosphorylation, we elected to further dissect the potential insulin signaling regulatory role of the previously uncharacterized protein PALD (KIAA1274, paladin). First, we confirmed the high throughput immunoassay results by examining AKT phosphorylation at S473 by Western blotting, as well as extending the analysis to the AKT T308 site (Figure 4A). PALD overexpression resulted in diminished insulin-stimulated AKT phosphorylation at both S473 and T308 at all time points tested (Figure 4A).

To further establish the biological relevance of PALD in insulin signaling, we performed PALD knockdown in rat liver H4IIE cells containing an integrated G6Pase-Luc reporter (H4IIE-G6Pase, Figure 4B). One of the two siRNAs (rPALD2) that were tested effectively knocked down rPALD mRNA expression levels (∼80%, Figure 4B inset) and, as expected, increased insulin sensitivity measured by reporter activity. To further investigate the relationship between PALD expression and insulin-simulated AKT phosphorylation and to expand the range of insulin-responsive cell lines tested, we electroporated the mouse myoblast C2C12 cell line with a mPALD siRNA Smartpool (an equimolar pool of 4 individual siRNAs), and separately with two individual siRNAs from the pool, achieving 60–70% reduction of mPALD mRNA levels and increasing insulin-induced AKT phosphorylation at both S473 and T308 (Figure 4C). These results, obtained in multiple cellular contexts using both reporter activation and AKT phosphorylation status, strongly suggest a role for PALD in insulin-induced AKT phosphorylation.

Because overexpression of PALD resulted in the inhibition of both S473 and T308 phosphorylation on AKT, we hypothesized that PALD could be targeting an upstream component in the insulin signaling pathway, including the insulin receptor (IR). We stripped the membrane used in Figure 4A and re-probed with an anti-insulin receptor β subunit antibody and a phosphotyrosine-specific antibody targeting Y1162/Y1163 of IR. Forced expression of PALD yielded a reduction of phosphorylated Y1162/Y1163 that correlated with a decreased overall abundance of the mature IR β subunit (Figure 4D), with both bands exhibiting a faster migration on the gel. These observations are unlikely due to inconsistent loading, since the level of total AKT is fairly constant (Figure 4A). Moreover, PALD-mediated down regulation of IR was likely not simply an artifact of overexpression since PALD depletion by siRNA enhanced IR abundance and Y1162/Y1163 phosphorylation levels with a corresponding augmentation in insulin-induced AKT activation (i.e., T308 phosphorylation, Figure 4E). Together these results implicate a role for PALD as a negative regulator of insulin signaling.

## Discussion

In the present study, we successfully integrated an arrayed cDNA functional profiling platform with an insulin responsive reporter that targeted the FOXO1A pathway to search for novel negative modulators of insulin signaling. Although the screen was biased toward a particular arm of the insulin signaling pathway, we identified proteins that act upstream in the pathway. In particular, we found that PALD inhibits insulin-stimulated AKT phosphorylation and this correlates with a decrease in IR abundance.

PALD (KIAA1274) was identified in large-scale cDNA sequencing efforts [13]. As such, relatively little is known about its function. The cDNA encodes a protein of 856 amino acids with two phosphatase signature motifs (CXXGXGR) and an N-terminal myristoylation site. The regions containing the phosphatase signature motifs are related to one another, but are only distantly related to the protein tyrosine phosphatase family [14]. The protein exhibits 41% amino acid identity across vertebrate species (data not shown). EST (www.ncbi.nlm.nih.gov/UniGene) and gene expression (biogps.gnf.org) databases show that it is expressed tissue specifically, with highest expression in lung and brown adipose tissue and significant expression in brain and embryonic tissue and in C2C12 cells. The human protein atlas (proteinatlas.org) shows a wider expression pattern with three different forms of the protein visible by western blot [15]. It is therefore tempting to speculate that PALD influences insulin signaling by dephosphorylating IR upon insulin-mediated autophosphorylation, leading to IR turnover. To date, however, we have been unable to detect phosphatase activity with full-length or truncated expression forms of PALD (data not shown) and so the precise mechanism by which PALD modulates IR abundance remains to be determined. Nevertheless, to our knowledge, this is the first functional characterization of PALD in insulin signaling or any other pathway. *Pald* mouse knockouts, which are being generated, should further clarify PALD's role and necessity in mediating insulin signaling.

Other primary screen hits represent a diverse array of biochemical functions and include many known inhibitors of insulin signaling, such as phosphatase and tensin homolog (PTEN), which has been shown to negatively modulate insulin signaling by both gain-of-function and loss-of-function studies [4], [5], PI(3)K p85 β subunit (PIK3R2), whose concentration is critical in regulating PI(3)K activity [12], [16], and phosphoprotein enriched in astrocytes 15 (PEA15), which is up-regulated in type 2 diabetes and whose transgenic expression causes diabetes by affecting both insulin sensitivity and insulin secretion [17], [18]. The identification of these well-characterized insulin signaling modulators as well as a number of additional hits previously linked to this pathway not only served as effective internal positive controls but also support the premise of our primary screening approach.

Whereas the primary assay merely identified known and novel inhibitors of insulin signaling, we applied a series of secondary assays in order to shed further light on the role such inhibitors may play. NCK2 for example, was previously implicated in insulin signaling due to its interaction with insulin receptor substrate-1 (IRS-1) [19]. Here we found that NCK2 inhibits insulin signaling and does so by repressing insulin-induced AKT phosphorylation. DOK2 has also been implicated in insulin signaling by virtue of its similarity to IRS family members [20]; we now provide functional evidence for a role for DOK2 as an inhibitor of insulin action.

Members of two distinct phosphatase families, the receptor type protein tyrosine phosphatase (PTPR) family (e.g. PTPRE and PTPRA) and the dual specificity phosphatase (DUSP) family (e.g. DUSP1, 4, 6, 7, and 10, these are also known as MAP kinase phosphatases, MKPs), were prominent among our hits and certain family members have previously been implicated in insulin signaling. Notably, in addition to DUSP6 (MKP3) and DUSP7 (MKPX), which were previously shown to overcome insulin's repression of the PEPCK promoter via an unknown mechanism [21], [22], we identified DUSP1 (MKP1), DUSP4 (MKP2), and DUSP10 (MKP5) as potential inhibitors of insulin signaling. We also further demonstrated that some of these phosphatases (DUSP 1, 4, 7, 10, PTPRE v2, and PTPRR v2) affect insulin-regulated FOXO1A activity without inhibiting AKT phosphorylation. The fact that several of the DUSPs inhibited insulin regulated FOXO1A activity independent of AKT phosphorylation at S473 (Figure 2B and 3A) indicates that the MAPK pathway plays a role in regulating FOXO1A activity. Such a role has recently been demonstrated for ERK and p38MAPK, which phosphorylate FOXO1 at sites distinct from those phosphorylated by AKT [23]. Phosphorylation of FOXO1 at the ERK sites promotes its interaction with the transcription factor ETS and potentiates ETS's ability to activate transcription [23].

In addition to PALD, a number of the confirmed screen hits have not previously been implicated in insulin signaling and are therefore putative novel inhibitors of insulin signaling. For example, active BCR related gene (ABR), a GTPase associated protein that exhibits intrinsic GAP and GEF activities, was previously demonstrated to participate in postnatal cerebellar development [24]. ABR overexpression potently inhibited insulin's effect on FOXO1A-mediated transcription and AKT phosphorylation (Figure 2B and 3A). Although sharing 68% amino acid sequence identity to ABR [25], [26], BCR overexpression had only a minor inhibitory effect on insulin-induced AKT phosphorylation (data not shown). It has recently been reported that both BCR and ABR are GAPs for Rac and loss of either ABR or BCR leads to abnormal macrophage morphology and motility [27]. Consistently, we find that overexpression of exogenous ABR leads to cells with fewer protrusions (data not shown) and this many explain in part its effects on insulin signaling.

Another noteworthy hit not previously linked to insulin signaling was the adaptor SAM and SH3 domain containing 1 (SASH1). Recent work has led to the prediction that SASH1 may function as a tumor suppressor and play a significant role in breast cancer and lung cancer [28]. Our studies showed that SASH1 can also significantly inhibit insulin's ability to repress FOXO1A-mediated transcription by inhibiting insulin-induced AKT phosphorylation (Figure 2B and 3A). Since insulin signaling via the PI(3)K-AKT pathway also plays a role in cell proliferation, our results may have pinpointed the mechanism by which SASH1 regulates cell proliferation.

Although the overall biological significance of our screen results will require further studies for each individual hit, the combination of different pathway assays we employed for initial follow-up has already provided significant preliminary validation and revealed interesting mechanistic leads for many of the screen hits. In conclusion, the present data strongly illustrate the applicability of arrayed genome-wide screening technologies to identifying novel modulators of insulin signaling.

## Materials and Methods

### Materials

Anti-phospho-insulin receptor (Tyr 1162/1163) antibody was purchased from Calbiochem (San Diego, CA). Anti-insulin receptor β subunit antibodies were acquired from Santa Cruz Biotechnology Inc. (Santa Cruz, CA) and BD Transduction Laboratories (San Diego, CA). Affinity purified antibodies against total AKT and phosphorylated AKTs (Thr 308 and Ser 473) were obtained from Cell Signaling Technology Inc. (Beverly, MA). Anti-flag antibody was obtained from Sigma (St. Louis, MO). The OriGene TrueClone Collection of human full-length cDNA clones was purchased from OriGene Technologies (Rockville, MD). For rat PALD knockdown studies, Negative Control siRNA Alexa Fluor 546 (5′-TTCTCCGAACGTGTCACGT-3′), rPALD-1 (5′-GCAGCCCACCTACAGATAT-3′), and rPALD-2 (5′-CCTCTTTAATGCCTACCTT-3′) were purchased from Qiagen (Valencia, CA). For mouse PALD knockdown studies, the following siRNAs were purchased from Dharmacon: siCONTROL Non-Targeting siRNA #1 (5′-TAGCGACTAAACACATCAA-3′), mPALD SMARTpool, mPALD-01 (5′-GGCCTTACCTACTGCCGTA-3′), and mPALD-02 (5′-GAGATAATGTGTACCACGT-3′).

### Constructs

The G6Pase-Luc reporter was constructed by cloning a ∼1.7 kbp fragment (−1631→+25) of the 5′-UTR of the human G6Pase catalytic subunit into the pGL3-Basic Vector (Promega, Madison, WI). The IRE-4U pGL3 reporter was constructed by concatamerizing four insulin responsive elements, each of which contains three FOXO binding motifs, from the human G6P catalytic subunit 5′-UTR (−185→−157).

### High-Throughput cDNA screen

An arrayed library of 18,441 human full-length cDNAs from the OriGene TrueClone Collection was screened by high-throughput reverse transfection of Chinese Hamster Ovary (CHO-K1) cells. Briefly, transfection mixture containing Opti-MEM I (Invitrogen, Carlsbad, CA), G6Pase-Luc reporter (2 ng/well), human FOXO1A (50 ng/well), and FuGENE 6 (Roche, Indianapolis, IN) was dispensed into 384-well plates pre-spotted with individual cDNAs (∼40 ng/well) and incubated at room temperature for 1 hour. CHO-K1 cells, cultured in DMEM/F12 supplemented with 10% FBS and penicillin/streptomycin, were then added (11,000 cells/well) and the 384-well plates were incubated overnight at 37°C, 5% CO_2_. Subsequently, cells were starved in serum-free DMEM/F12 for 8 hours prior to treatment with a mixture containing 500 nM human recombinant insulin (Sigma-Aldrich, St. Louis, MO) and 2 mM dibutyryl-cAMP (Roche, Indianapolis, IN). Approximately 24 hours after treatment, Bright-Glo Reagent (Promega, Madison, WI) was added to each well and luminescence readings were acquired using an Acquest microplate reader (Molecular Devices, Sunnyvale, CA). Follow-up assays with and without insulin were performed similarly except that a conventional FuGENE 6-mediated forward transfection protocol (cells first) was followed using either the G6Pase-Luc reporter or IRE-4U reporter.

### Phospho (Ser473)/Total AKT Multiplex Immunoassay

Phospho (Ser 473) and total AKT levels in cell lysates following transient transfection of selected screen hits were measured using the MSD MULTI-SPOT Assay System (Meso Scale Discovery, Gaithersburg, MD). Briefly, 96-well plates pre-seeded with COS7 cells to be ∼80% confluent were transfected with selected cDNAs (150 ng/well) using the TransIT-COS Transfection Kit (Mirus, Madison, WI). The transfected COS7 cells were cultured in DMEM supplemented with 10% heat-inactivated FBS at 37°C and 5% CO_2_ for 16–24 hours prior to replacing the growth medium with serum-free DMEM. At various time-points following a minimum of 6 hours in serum-free conditions, selected wells were treated with human recombinant insulin (50 nM final concentration). At the end of the time-course, approximately 20–32 hours following initiation of the serum-free incubation step, cell lysates were prepared on ice using MSD Complete Lysis Buffer. Cell lysates were transferred to pre-blocked MSD MULTI-SPOT 96-well plates and incubated with shaking for 1–2 hours at room temperature, washed several times, incubated with MSD SULFO-TAG Detection Antibody with shaking for an additional 1-2 hours at room temperature, washed several times, and analyzed in MSD Read Buffer T using an MSD Sector Imager 6000. Fold-repression of insulin-induced AKT phosphorylation for each of the assayed screen hits was determined by calculating the ratio of phospho AKT to total AKT (pAKT/tAKT) for the vector control, pCMV6-XL5, and dividing it by the pAKT/tAKT ratio obtained for each individual cDNA.

### Quantitative PCR

Quantitative PCR reactions were performed using the SuperScript III Platinum One-Step qRT-PCR Kit (Invitrogen, Carlsbad, CA) and an ABI PRISM 7900HT Sequence Detection System (Applied Biosystems, Foster, CA) according to manufacturer's protocol. The qPCR probe and primers sequences for rat PALD were as follows: probe, 5′-TGCCTGTGCAGAGTTACATGACCT-3′; forward primer, 5′-GAGGTGGATCGAGCCATCA-3′; reverse primer 5′- TGGTTTTTGAGGAGCTCTTCCT-3′. The qPCR probe and primers sequences for mouse PALD were as follows: probe, 5′- TGCGGCGTTGGACATTGTC-3′; forward primer, 5′- GCCACCACGTGAAGAAGGA-3′; reverse primer 5′- GAAGGTGGTAGTGCATAGGTGTCA-3′.

### siRNA Transfection

300 pmoles of siRNAs targeting mouse PALD or rat PALD were electroporated into 2×10^6^ C2C12 or H4IIE cells using an Amaxa Nucleofector system (Amaxa, Gaithersburg, MD) according to manufacturer's protocol. Cells were subsequently cultured for 48–72 hours before insulin stimulation and harvesting the cells for downstream applications.

### Western Blot Analysis

Briefly, proteins were extracted from transfected COS7 cells (prepared as detailed above) using MSD Complete Lysis Buffer (Meso Scale Discovery, Gaithersburg, MD) or from electroporated C2C12 cells using Triton X lysis buffer comprising 50 mM Tris-HCl, pH 7.5, 100 mM NaCl, 1% Triton X-100, 10% glycerol, 1 mM EDTA, and 0.1 µg/ml Calyculin A (Cell Signaling Technology, Beverly, MA), and protease inhibitor and phosphatase inhibitor cocktails (Sigma-Aldrich, St. Louis, MO). Total cellular proteins were separated by 10% or 8% Tris-glycine SDS/PAGE (Invitrogen, Carlsbad, CA) and transferred to nitrocellulose membranes. Proteins were detected with primary antibodies and horseradish peroxidase-conjugated secondary antibodies by using the ECL-Plus kit (GE Healthcare, United Kingdom).

## Supporting Information

Figure S1 CHO-K1 cells are responsive to insulin stimulation and express an insignificant amount of endogenous FOXO1A transcription factor. (a) CHO-K1 cells were treated with 100 nM insulin for the times indicated and analyzed for AKT phosphorylation and total IR expression by western blot. (b) Expression level of FOXO1A in CHO-K1 cells. Human FOXO1A cDNA and the vector control were transfected into CHO-K1 cells and allowed to express for 48 hours. Cell extracts were taken and analyzed by Western blotting using anti-FKHR (Cell Signaling Technology, Beverly, MA) as primary antibody. (c) G6Pase-Luc reporter is inactive in CHO-K1 cells without exogenous expression of FOXO1A. CHO-K1 cells were co-transfected with 2 ng or 5 ng of G6Pase-Luc and 50 ng or 75 ng of α-tubulin (negative control) or FOXO1A. Insulin and cAMP stimulations were performed as described in Methods.
(0.07 MB PDF)

Figure S2 Phospho-(Ser473)/Total AKT Multiplex Immunoassay Results. As detailed in the legend for Table S3, up to three independent experiments were carried out for each cDNA whose pAKT and tAKT levels were simultaneously measured as detailed in the Methods. For comparative purposes, the computed average pAKT/tAKT ratios ± the standard deviations for the indicated time-points from each independent experiment performed on an individual cDNA (in yellow; listed in alphabetical order) are plotted relative to the corresponding microplate values obtained with the negative vector control, pCMV6-XL5 (in red), and the positive control, Grb10 (in blue). n.d., not determined.
(0.69 MB PDF)

Table S1 Summary of the first cell-based follow-up assay. Data obtained and analyzed as described in figure 2a.
(0.07 MB PDF)

Table S2 Summary of the second cell-based follow-up assay. Data obtained and analyzed as described in figure 2b.
(0.05 MB PDF)

Table S3 Phospho-(Ser473)/Total AKT Multiplex Immunoassay Data. Shown here are the raw pAKT and tAKT data sets as well as the calculated pAKT/tAKT ratios for each of the selected screen hits (listed in alphabetical order) that were assayed as detailed in the Methods. The four independent experiments that were performed are color-coded with additional specific time-course differences indicated in the table heading. The “0” time-point corresponds to no insulin treatment while the insulin (50 nM) treatment times are indicated in minutes (m) and hours (h). Generally, for each time- point examined two or three replicates were performed per experiment and up to three independent experiments were carried out for each assayed cDNA. For comparative purposes, the computed average pAKT/tAKT ratios ± the standard deviations for the indicated time-points from each independent experiment performed on an individual cDNA are plotted in Figure S2 relative to the corresponding microplate values obtained with the negative vector control, pCMV6-XL5, and the positive control, Grb10.
(0.05 MB PDF)
