# Supplementary material for: Negative Regulators of Insulin Signaling Revealed in a Genome-Wide Functional Screen
Source: PLoS One. 2009 Sep 3;4(9):e6871. doi: 10.1371/journal.pone.0006871 (PMC2731165; doi:10.1371/journal.pone.0006871)
Supplement: Figure S1 — CHO-K1 cells are responsive to insulin stimulation and express an insignificant amount of endogenous FOXO1A transcription factor. (a) CHO-K1 cells were treated with 100 nM insulin for the times indicated and analyzed for AKT phosphorylation and total IR expression by western blot. (b) Expression level of FOXO1A in CHO-K1 cells. Human FOXO1A cDNA and the vector control were transfected into CHO-K1 cells and allowed to express for 48 hours. Cell extracts were taken and analyzed by Western blotting using anti-FKHR (Cell Signaling Technology, Beverly, MA) as primary antibody. (c) G6Pase-Luc reporter is inactive in CHO-K1 cells without exogenous expression of FOXO1A. CHO-K1 cells were co-transfected with 2 ng or 5 ng of G6Pase-Luc and 50 ng or 75 ng of α-tubulin (negative control) or FOXO1A. Insulin and cAMP stimulations were performed as described in Methods. (0.07 MB PDF) [file pone.0006871.s001.pdf]

Figure S1

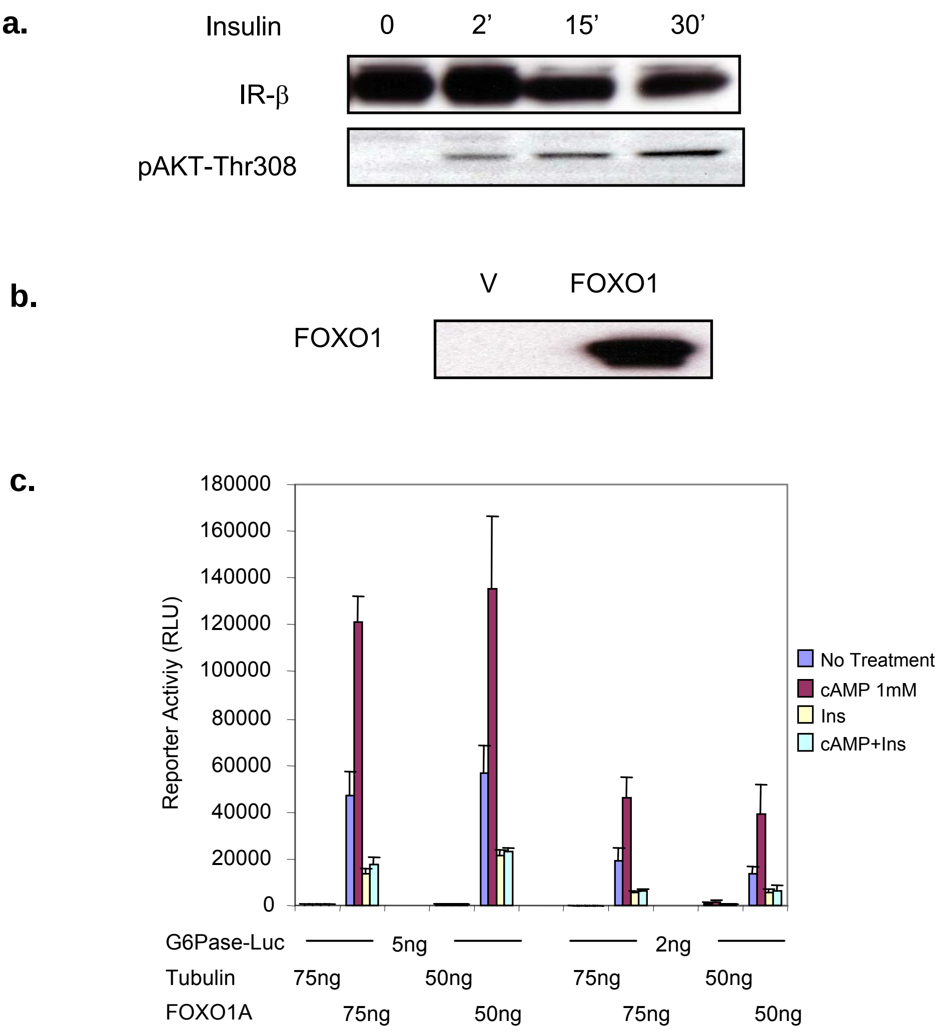

Fig S1. CHO-K1 cells are responsive to insulin stimulation and express an insignificant amount of endogenous FOXO1A transcription factor (a) CHO-K1 cells were treated with 100 nM insulin for the times indicated and analyzed for AKT phosphorylation and total IR expression by western blot. (b) Expression level of FOXO1A in CHO-K1 cells. Human FOXO1A cDNA and the vector control were transfected into CHO-K1 cells and allowed to express for 48 hours. Cell extracts were taken and analyzed by Western blotting using anti-FKHR (Cell Signaling Technology, Beverly, MA) as primary antibody. (c) G6Pase-Luc reporter is inactive in CHO-K1 cells without exogenous expression of FOXO1A. CHO-K1 cells were co-transfected with 2 ng or 5 ng of G6Pase-Luc and 50ng or 75ng of α-tubulin (negative control) or FOXO1A. Insulin and cAMP stimulations were performed as described in Methods.
