# Supplementary material for: Negative Regulators of Insulin Signaling Revealed in a Genome-Wide Functional Screen
Source: PLoS One. 2009 Sep 3;4(9):e6871. doi: 10.1371/journal.pone.0006871 (PMC2731165; doi:10.1371/journal.pone.0006871)
Supplement: Figure S2 — Phospho-(Ser473)/Total AKT Multiplex Immunoassay Results. As detailed in the legend for Table S3, up to three independent experiments were carried out for each cDNA whose pAKT and tAKT levels were simultaneously measured as detailed in the Methods. For comparative purposes, the computed average pAKT/tAKT ratios ± the standard deviations for the indicated time-points from each independent experiment performed on an individual cDNA (in yellow; listed in alphabetical order) are plotted relative to the corresponding microplate values obtained with the negative vector control, pCMV6-XL5 (in red), and the positive control, Grb10 (in blue). n.d., not determined. (0.69 MB PDF) [file pone.0006871.s002.pdf]

Figure S2

Experiment 1	Experiment 2	Experiment 3/Experiment 4
n.d.	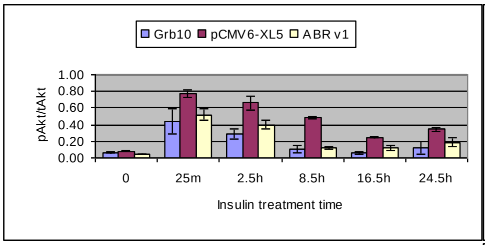	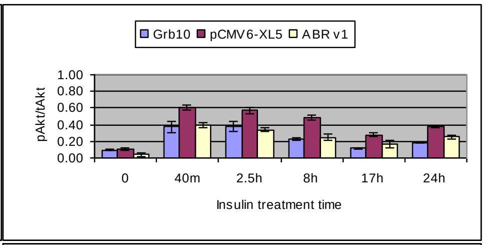
n.d.	n.d.	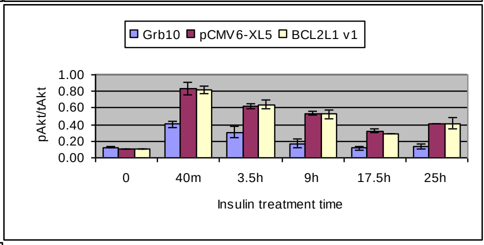
n.d.	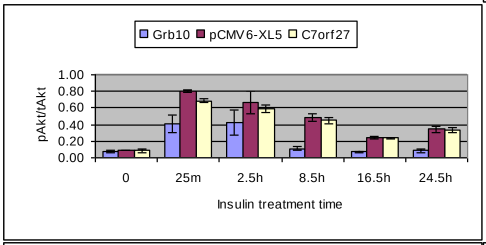	n.d.
n.d.	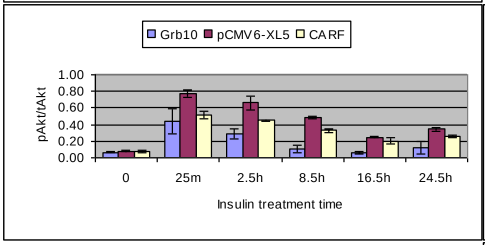	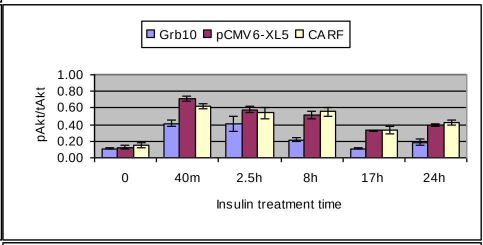
n.d.	n.d.	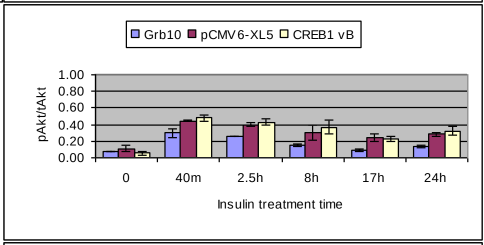
n.d.	n.d.	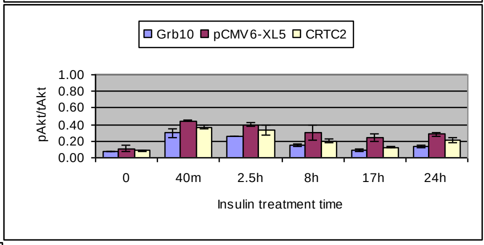
n.d.	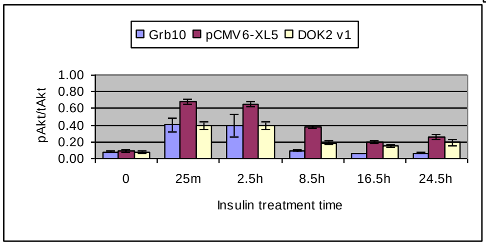	n.d.

Figure S2

Experiment 1 **Experiment 2** **Experiment 3/Experiment 4**

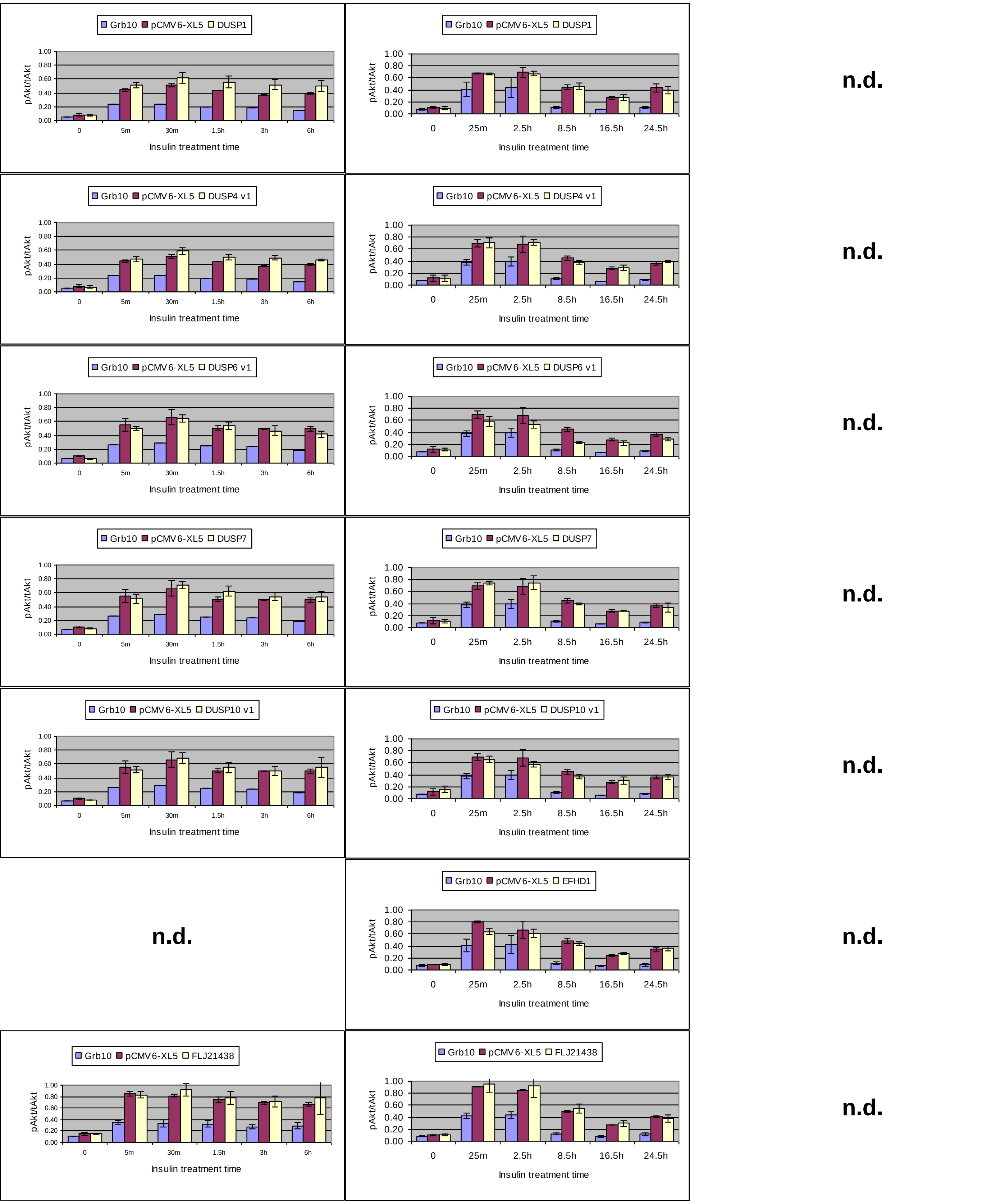

Figure S2

Experiment 1	Experiment 2	Experiment 3/Experiment 4
n.d.	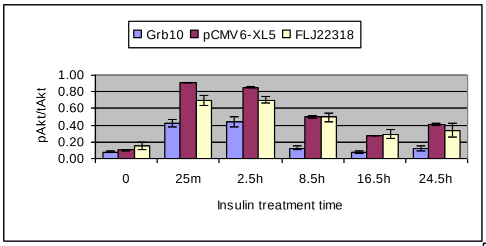	n.d.
n.d.	n.d.	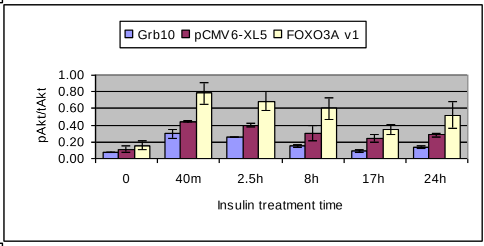
n.d.	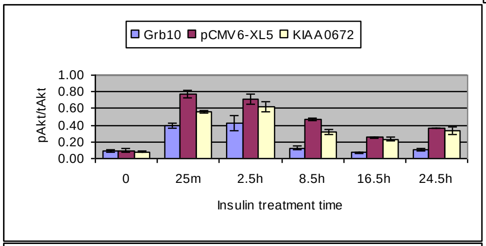	n.d.
n.d.	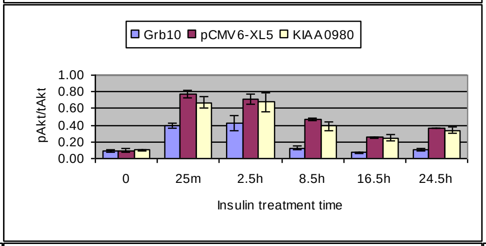	n.d.
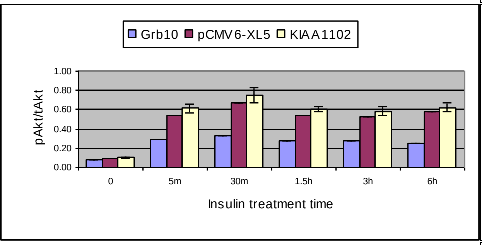	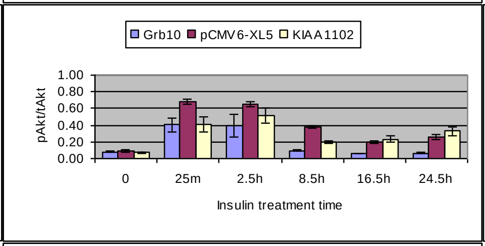	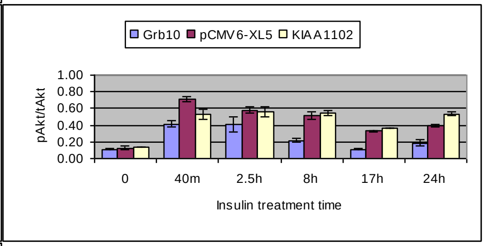
n.d.	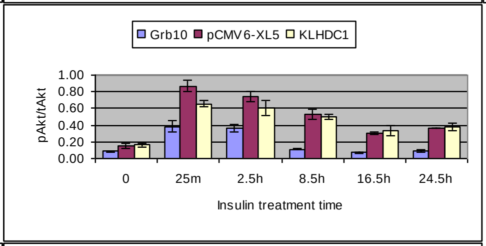	n.d.
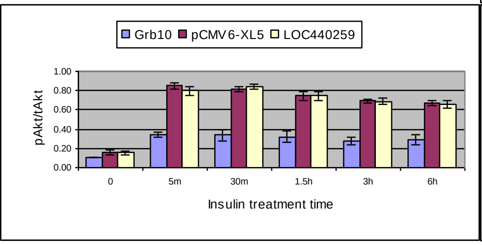	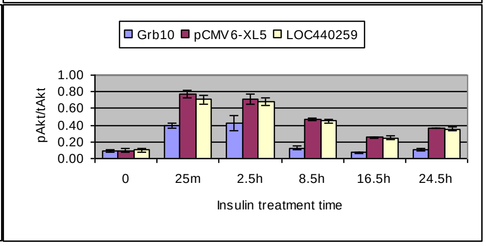	n.d.

Figure S2

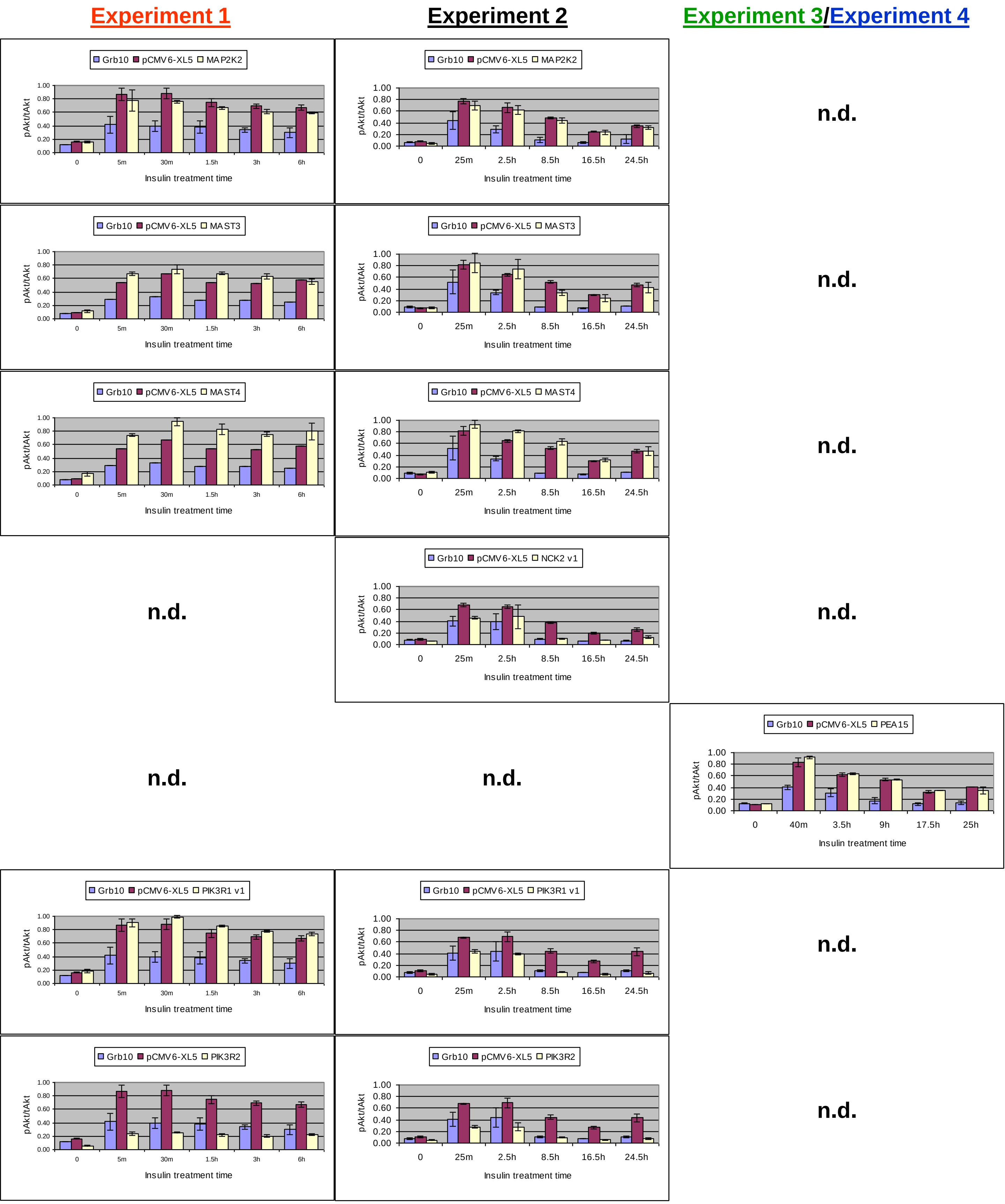
Experiment 1
Experiment 2
Experiment 3/Experiment 4
Grb10 pCMV6-XL5 MAP2K2
pAkt/tAkt
1.00 0.80 0.60 0.40 0.20 0.00
0 5m 30m 1.5h 3h 6h
Insulin treatment time
Grb10 pCMV6-XL5 MAP2K2
pAkt/tAkt
1.00 0.80 0.60 0.40 0.20 0.00
0 25m 2.5h 8.5h 16.5h 24.5h
Insulin treatment time
n.d.
Grb10 pCMV6-XL5 MAST3
pAkt/tAkt
1.00 0.80 0.60 0.40 0.20 0.00
0 5m 30m 1.5h 3h 6h
Insulin treatment time
Grb10 pCMV6-XL5 MAST3
pAkt/tAkt
1.00 0.80 0.60 0.40 0.20 0.00
0 25m 2.5h 8.5h 16.5h 24.5h
Insulin treatment time
n.d.
Grb10 pCMV6-XL5 MAST4
pAkt/tAkt
1.00 0.80 0.60 0.40 0.20 0.00
0 5m 30m 1.5h 3h 6h
Insulin treatment time
Grb10 pCMV6-XL5 MAST4
pAkt/tAkt
1.00 0.80 0.60 0.40 0.20 0.00
0 25m 2.5h 8.5h 16.5h 24.5h
Insulin treatment time
n.d.
n.d.
Grb10 pCMV6-XL5 NCK2 v1
pAkt/tAkt
1.00 0.80 0.60 0.40 0.20 0.00
0 25m 2.5h 8.5h 16.5h 24.5h
Insulin treatment time
n.d.
n.d.
n.d.
Grb10 pCMV6-XL5 PEA15
pAkt/tAkt
1.00 0.80 0.60 0.40 0.20 0.00
0 40m 3.5h 9h 17.5h 25h
Insulin treatment time
Grb10 pCMV6-XL5 PIK3R1 v1
pAkt/tAkt
1.00 0.80 0.60 0.40 0.20 0.00
0 5m 30m 1.5h 3h 6h
Insulin treatment time
Grb10 pCMV6-XL5 PIK3R1 v1
pAkt/tAkt
1.00 0.80 0.60 0.40 0.20 0.00
0 25m 2.5h 8.5h 16.5h 24.5h
Insulin treatment time
n.d.
Grb10 pCMV6-XL5 PIK3R2
pAkt/tAkt
1.00 0.80 0.60 0.40 0.20 0.00
0 5m 30m 1.5h 3h 6h
Insulin treatment time
Grb10 pCMV6-XL5 PIK3R2
pAkt/tAkt
1.00 0.80 0.60 0.40 0.20 0.00
0 25m 2.5h 8.5h 16.5h 24.5h
Insulin treatment time
n.d.

Figure S2

Experiment 1	Experiment 2	Experiment 3/Experiment 4

n.d.

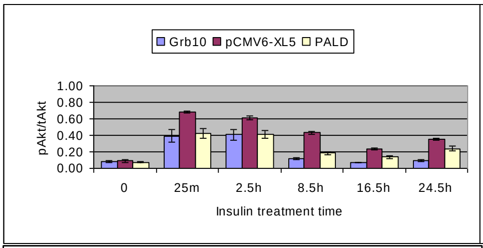

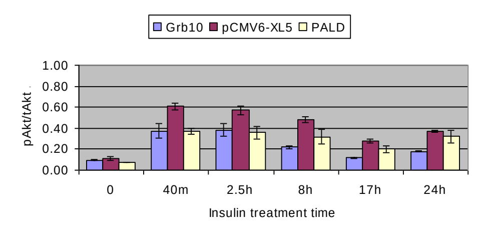

n.d.

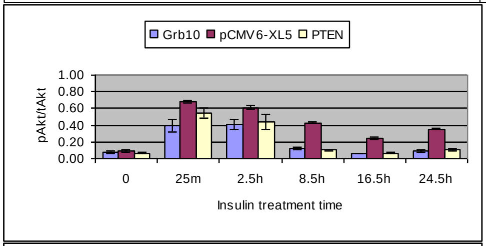

n.d.

n.d.

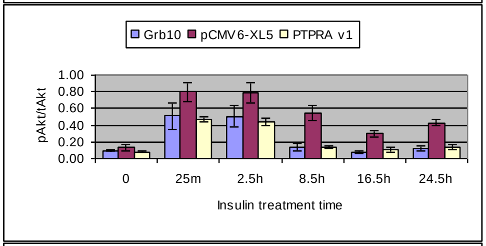

n.d.

n.d.

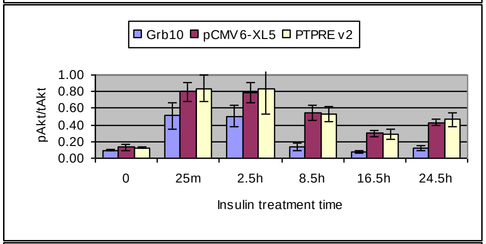

n.d.

n.d.

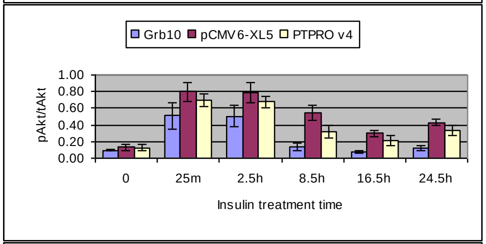

n.d.

n.d.

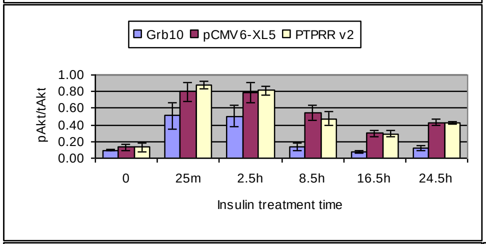

n.d.

n.d.

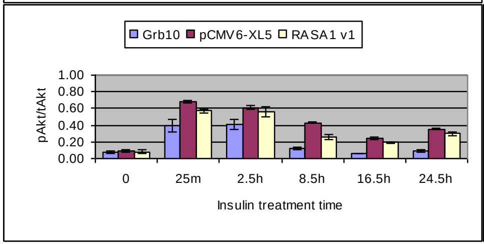

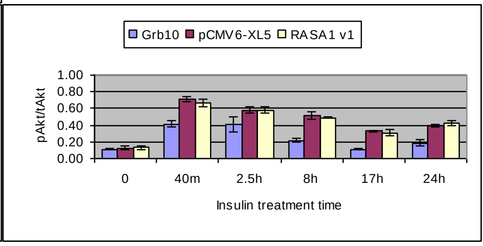

Figure S2

Experiment 1 | Experiment 2 | Experiment 3/Experiment 4

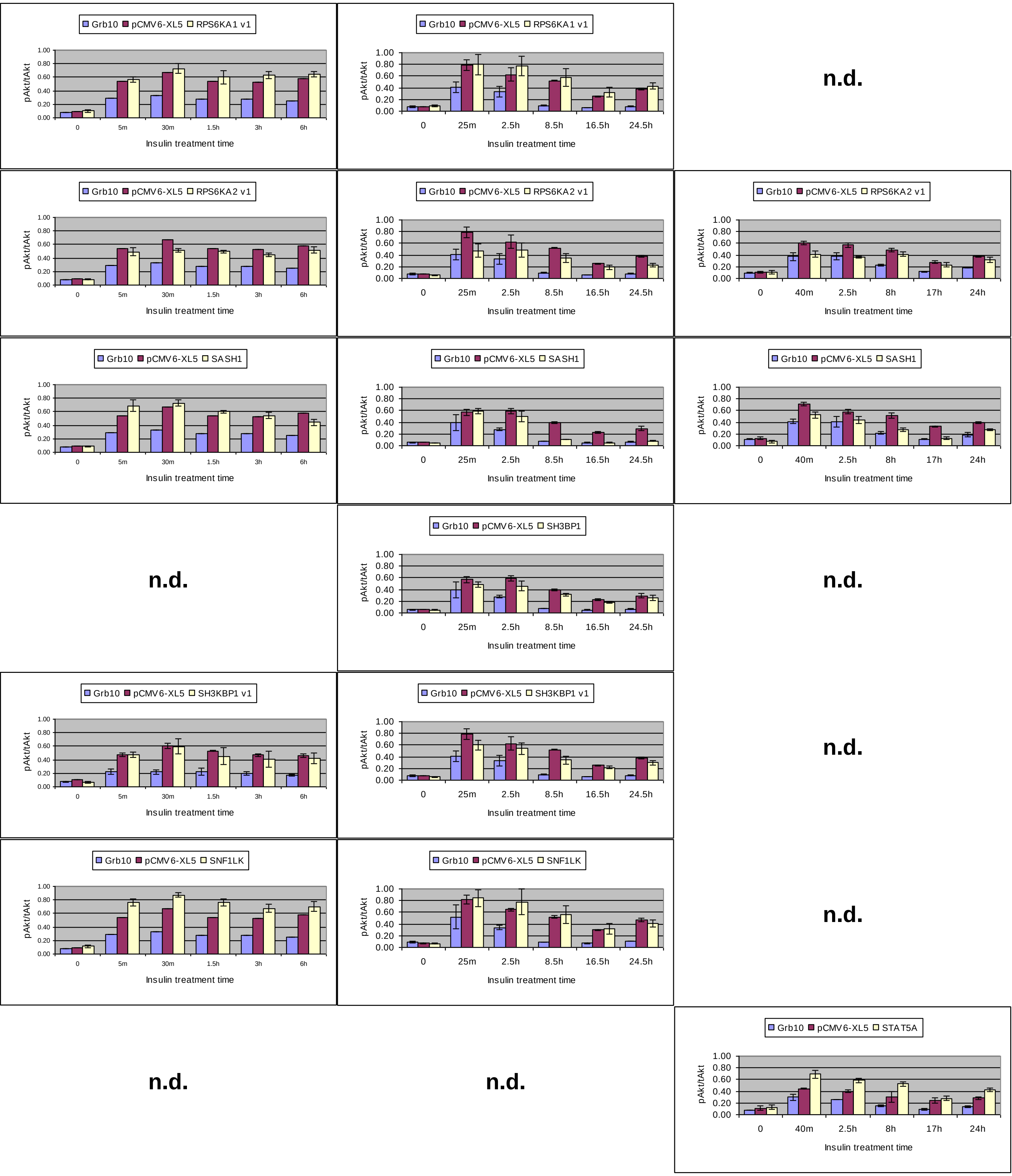

Figure S2

Experiment 1 | Experiment 2 | Experiment 3/Experiment 4

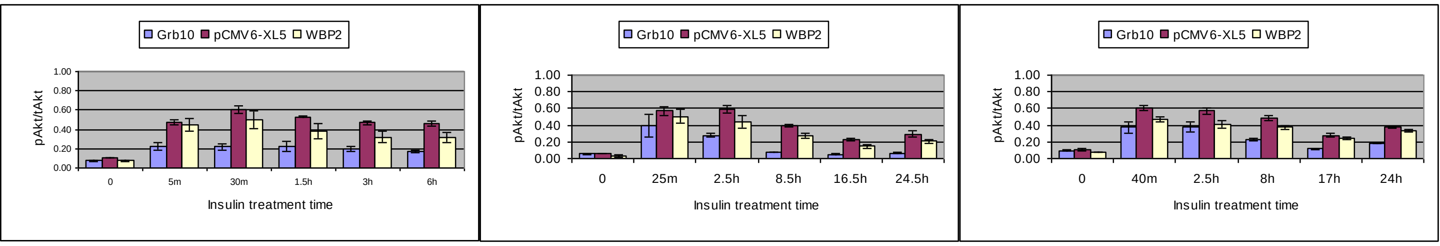

Figure S2. Phospho-(Ser473)/Total AKT Multiplex Immunoassay Results. As detailed in the legend for Table S3, up to three independent experiments were carried out for each cDNA whose pAKT and tAKT levels were simultaneously measured as detailed in the Methods. For comparative purposes, the computed average pAKT/tAKT ratios ± the standard deviations for the indicated time-points from each independent experiment performed on an individual cDNA (in yellow; listed in alphabetical order) are plotted relative to the corresponding microplate values obtained with the negative vector control, pCMV6-XL5 (in red), and the positive control, Grb10 (in blue). n.d., not determined.
