# Supplementary material for: Negative Regulators of Insulin Signaling Revealed in a Genome-Wide Functional Screen
Source: PLoS One. 2009 Sep 3;4(9):e6871. doi: 10.1371/journal.pone.0006871 (PMC2731165; doi:10.1371/journal.pone.0006871)
Supplement: Table S1 — Summary of the first cell-based follow-up assay. Data obtained and analyzed as described in figure 2a. (0.07 MB PDF) [file pone.0006871.s003.pdf]

Table S1. Summary of the first cell-based follow-up assay. Data obtained and analyzed as described in the legend to figure 2a.

Symbol+variant	Other Aliases:	Updated Accession	Ratio	Stdev	P val
ABR v1	MDB	NM_021962	0.31	0.04	0.0022
ATF1	TREB36	NM_005171	0.28	0.04	0.0064
BCL2L1 v1	BCL-XL/S, BCL2L, BCLX, Bcl-X, DKFZp781P2092, bcl-xL, bcl-xS	NM_138578	0.27	0.04	0.0222
DOK2 v1	p56DOK, p56dok-2	NM_003974	0.30	0.05	0.0071
DUSP1	CL100, HVH1, MKP-1, PTPN10	NM_004417	0.54	0.05	0.0001
DUSP10 v1	MKP-5, MKP5	NM_007207	0.36	0.06	0.0020
DUSP4 v1	HVH2, MKP-2, MKP2, TYP	NM_001394	0.52	0.12	0.0021
DUSP6 v1	MKP3, PYST1	NM_001946	0.63	0.06	0.0001
DUSP7	MKP-X, MKPX, PYST2	NM_001947	0.50	0.10	0.0008
FLJ21438	FLJ00087	XM_029084	0.27	0.03	0.0009
FOXO3A v1	AF6q21, DKFZp781A0677, FKHRL1, FKHRL1P2, MGC12739, MGC31925	NM_001455	0.46	0.13	0.0038
Grb10	RP23-119N24.1, 5730571D09Rik, AI325020, Meg1, mKIAA0207	NM_010345	0.70	0.09	0.0001
KIAA1274	RP11-710A11.1, PALD	NM_014431	0.27	0.05	0.0286
MAP2K2	MAPKK2, MEK2, MKK2, PRKMK2	NM_030662	0.24	0.07	0.0412
MAST3	KIAA0561	XM_038150	0.34	0.02	0.0001
MAST4	KIAA0303	XM_291141	0.29	0.04	0.0019
NCK2 v1	GRB4, NCKbeta	NM_003581	0.31	0.10	0.0233
PEA15	HMAT1, HUMMAT1H, MAT1, MAT1H, PEA-15, PED	NM_003768	0.31	0.09	0.0157
PIK3R1 v1	GRB1, p85-ALPHA	NM_181523	0.52	0.22	0.0162
PIK3R2	P85B, p85-BETA	NM_005027	0.62	0.08	0.0001
PTEN	BZS, MGC11227, MHAM, MMAC1, PTEN1, TEP1	NM_000314	0.35	0.04	0.0001
PTPRA v1	RP4-534B8.1, HEPTP, HLPR, HPTPA, HPTPalpha, LRP, PTPA, PTPRL2, R-PTP-alpha, RPTPA	NM_002836	0.24	0.04	0.0161
PTPRA v2	RP4-534B8.1, HEPTP, HLPR, HPTPA, HPTPalpha, LRP, PTPA, PTPRL2, R-PTP-alpha, RPTPA	NM_080840	0.33	0.04	0.0005
PTPRE v1	DKFZp313F1310, HPTPE, PTPE, R-PTP-EPSILON	NM_006504	0.55	0.07	0.0001
PTPRE v2	DKFZp313F1310, HPTPE, PTPE, R-PTP-EPSILON	NM_130435	0.50	0.11	0.0009
PTPRR v1	DKFZp781C1038, EC-PTP, PCPTP1, PTP-SL, PTPBR7, PTPRQ	NM_002849	0.29	0.05	0.0029
PTPRR v2	DKFZp781C1038, EC-PTP, PCPTP1, PTP-SL, PTPBR7, PTPRQ	NM_130846	0.30	0.07	0.0091
RASA1 v1	CMAVM, DKFZp434N071, GAP, PKWS, RASA, RASGAP, p120GAP	NM_002890	0.34	0.04	0.0003
RPS6KA1 v1	HU-1, MAPKAPK1A, RSK, RSK1, S6K-alpha 1	NM_002953	0.42	0.08	0.0006
RPS6KA2 v1	HU-2, MAPKAPK1C, RSK, RSK3, S6K-alpha, S6K-alpha2, p90-RSK2, pp90RSK3	NM_021135	0.31	0.10	0.0226
SASH1	KIAA0790, RP3-323M4.1, dJ323M4.1	NM_015278	0.31	0.03	0.0002
SH3BP1		NM_018957	0.29	0.07	0.0110
SH3KBP1 v1	CIN85, MIG18	NM_031892	0.31	0.05	0.0011
WBP2	MGC18269, WBP-2	NM_012478	0.21	0.01	0.0122
C7orf27	MGC22916	NM_152743	0.23	0.02	0.0440
CREB1 vB	CREB, MGC9284	NM_134442	0.37	0.03	0.0001
CRTC2	RP11-422P24.6, TORC2	NM_181715	0.34	0.05	0.0005
FLJ22318	DKFZp434K0926	NM_022762	0.24	0.04	0.0132
KIAA0672		NM_014859	0.27	0.04	0.0030
KIAA0980	RP4-691N24.1, FLJ11792, KIAA0980, NLP, dJ691N24.1	NM_025176	0.24	0.05	0.0214
KIAA1102	DKFZp686B2470, DKFZp781I1455, MGC72127	NM_014988	0.25	0.07	0.0418
KLHDC1	MST025	NM_172193	0.21	0.01	0.0125
LOC440259		XM_496056	0.23	0.03	0.0107
PTPRO v4	GLEPP1, PTP-U2, PTPU2	NM_030668	0.29	0.06	0.0063
SNF1LK	MSK, SIK	NM_173354	0.30	0.05	0.0020
STAT5A	MGF, STAT5	NM_003152	0.24	0.06	0.0380
K-ALPHA-1	α-tubulin	NM_006082	0.15	0.03	1.0000
ANKRD44	MGC21968, MGC70444	NM_153697	0.19	0.05	0.2016
C10orf30	FLJ40283, MGC35247	NM_152751	0.13	0.03	0.4023
C18orf25 v1	ARKL1, MGC12909, MGC87799	NM_145055	0.21	0.03	0.3406
CARF	FLJ20036	NM_017632	0.27	0.09	0.1382
DACH2	RP11-345E19.1, FLJ31391	NM_053281	0.17	0.05	0.5791
DEF6	IBP	NM_022047	0.15	0.02	0.9433
DGKZ v2	DAGK5, DAGK6, DGK-ZETA, hDGKzeta	NM_003646	0.20	0.04	0.1007
DUSP5	DUSP, HVH3	NM_004419	0.33	0.15	0.0538
EFHD1	DKFZp781H0842, FLJ13612, MST133, MSTP133, PP3051	NM_025202	0.26	0.08	0.1234
empty			0.15	0.02	0.7485
FAM49A	DKFZP566A1524, FLJ11080	NM_030797	0.21	0.03	0.4345
FLJ10159		NM_018013	0.17	0.01	0.2022
FLJ13841		NM_024702	0.19	0.03	0.0835
FLJ22965		NM_022101	0.17	0.03	0.2653
FLJ34187		AK091506	0.20	0.04	0.1166
GPATC4 v2		NM_182679	0.11	0.02	0.0718
ITPK1	ITRPK1	NM_014216	0.20	0.05	0.1394
LOC150223 v2		NM_001017965	0.20	0.08	0.2529

Table S1 (cont). Summary of the first cell-based follow-up assay. Data obtained and analyzed as described in the legend to figure 2a.

Symbol+variant	Other Aliases:	Updated Accession	Ratio	Stdev	P val
MICAL-L1	DKFZp686M2226, KIAA1668, MIRAB13	NM_033386	0.19	0.04	0.1195
NUDCD3	KIAA1068	NM_015332	0.19	0.05	0.1953
pCMV6-XL5		AF067196	0.19	0.03	0.0924
POGK	BASS2, KIAA1513, KIAA15131, LST003	NM_017542	0.15	0.03	0.9482
SIAH1 v1	HUMSIAH, Siah-1, Siah-1a, hSIAH1	NM_003031	0.47	0.34	0.1035
SIRT2 v2	SIR2L, SIR2L2	NM_030593	0.16	0.03	0.5184
TTLL5	KIAA0998	NM_015072	0.16	0.03	0.6292
ZBTB9	MGC23166	NM_152735	0.14	0.03	0.7085
ZNF385	DKFZP586G1122, HZF, RZF, ZFP385	NM_015481	0.20	0.04	0.0757
