# Supplementary material for: Negative Regulators of Insulin Signaling Revealed in a Genome-Wide Functional Screen
Source: PLoS One. 2009 Sep 3;4(9):e6871. doi: 10.1371/journal.pone.0006871 (PMC2731165; doi:10.1371/journal.pone.0006871)
Supplement: Table S2 — Summary of the second cell-based follow-up assay. Data obtained and analyzed as described in figure 2b. (0.05 MB PDF) [file pone.0006871.s004.pdf]

Table S2. Summary of the second cell-based follow-up assay. Data obtained and analyzed as described in the legend to figure 2b.

Symbol+variant	Other Aliases:	Updated Accession	Ratio	Stdev	P val
ABR v1	MDB	NM_021962	0.83	0.20	0.0048
ATF1	TREB36	NM_005171	0.49	0.06	0.0392
BCL2L1 v1	BCL-XL/S, BCL2L, BCLX, Bcl-X, DKFZp781P2092, bcl-xL, bcl-xS	NM_138578	0.53	0.11	0.0359
DOK2 v1	p56DOK, p56dok-2	NM_003974	0.64	0.10	0.0074
DUSP1	CL100, HVH1, MKP-1, PTPN10	NM_004417	0.79	0.09	0.0013
DUSP10 v1	MKP-5, MKP5	NM_007207	0.89	0.09	0.0006
DUSP4 v1	HVH2, MKP-2, MKP2, TYP	NM_001394	0.67	0.25	0.0332
DUSP6 v1	MKP3, PYST1	NM_001946	0.85	0.17	0.0024
DUSP7	MKP-X, MKPX, PYST2	NM_001947	0.62	0.18	0.0273
FLJ21438	FLJ00087	XM_029084	0.60	0.06	0.0079
FOXO3A v1	AF6q21, DKFZp781A0677, FKHRL1, FKHRL1P2, MGC12739, MGC31925	NM_001455	0.71	0.06	0.0024
Grb10	RP23-119N24.1, 5730571D09Rik, AI325020, Meg1, mKIAA0207	NM_010345	0.99	0.17	0.0008
KIAA1274	RP11-710A11.1, PALD	NM_014431	0.54	0.07	0.0219
MAP2K2	MAPKK2, MEK2, MKK2, PRKMK2	NM_030662	0.50	0.06	0.0328
MAST3	KIAA0561	XM_038150	0.52	0.04	0.0226
MAST4	KIAA0303	XM_291141	0.52	0.10	0.0355
NCK2 v1	GRB4, NCKbeta	NM_003581	0.65	0.09	0.0059
PEA15	HMAT1, HUMMAT1H, MAT1, MAT1H, PEA-15, PED	NM_003768	0.63	0.14	0.0151
PIK3R1 v1	GRB1, p85-ALPHA	NM_181523	0.54	0.07	0.0201
PIK3R2	P85B, p85-BETA	NM_005027	0.60	0.15	0.0240
PTEN	BZS, MGC11227, MHAM, MMAC1, PTEN1, TEP1	NM_000314	0.61	0.08	0.0105
PTPRA v1	RP4-534B8.1, HEPTP, HLPR, HPTPA, HPTPalpha, LRP, PTPA, PTPRL2, R-PTP-alpha, RPTPA	NM_002836	0.53	0.09	0.0277
PTPRA v2	RP4-534B8.1, HEPTP, HLPR, HPTPA, HPTPalpha, LRP, PTPA, PTPRL2, R-PTP-alpha, RPTPA	NM_080840	0.65	0.18	0.0193
PTPRE v1	DKFZp313F1310, HPTPE, PTPE, R-PTP-EPSILON	NM_006504	0.58	0.07	0.0113
PTPRE v2	DKFZp313F1310, HPTPE, PTPE, R-PTP-EPSILON	NM_130435	0.73	0.17	0.0072
PTPRR v1	DKFZp781C1038, EC-PTP, PCPTP1, PTP-SL, PTPBR7, PTPRQ	NM_002849	0.64	0.07	0.0055
PTPRR v2	DKFZp781C1038, EC-PTP, PCPTP1, PTP-SL, PTPBR7, PTPRQ	NM_130846	0.59	0.06	0.0090
RASA1 v1	CMAVM, DKFZp434N071, GAP, PKWS, RASA, RASGAP, p120GAP	NM_002890	0.61	0.07	0.0079
RPS6KA1 v1	HU-1, MAPKAPK1A, RSK, RSK1, S6K-alpha 1	NM_002953	0.67	0.18	0.0164
RPS6KA2 v1	HU-2, MAPKAPK1C, RSK, RSK3, S6K-alpha, S6K-alpha2, p90-RSK2, pp90RSK3	NM_021135	0.80	0.16	0.0034
SASH1	KIAA0790, RP3-323M4.1, dJ323M4.1	NM_015278	0.53	0.07	0.0244
SH3BP1		NM_018957	0.58	0.10	0.0163
SH3KBP1 v1	CIN85, MIG18	NM_031892	0.63	0.10	0.0082
WBP2	MGC18269, WBP-2	NM_012478	0.51	0.04	0.0275
C7orf27	MGC22916	NM_152743	0.40	0.05	0.1579
CREB1 vB	CREB, MGC9284	NM_134442	0.40	0.05	0.1619
CRTC2	RP11-422P24.6, TORC2	NM_181715	0.40	0.08	0.1798
FLJ22318	DKFZp434K0926	NM_022762	0.36	0.09	0.3050
KIAA0672		NM_014859	0.48	0.15	0.0971
KIAA0980	RP4-691N24.1, FLJ11792, KIAA0980, NLP, dJ691N24.1	NM_025176	0.41	0.04	0.1320
KIAA1102	DKFZp686B2470, DKFZp781I1455, MGC72127	NM_014988	0.41	0.06	0.1440
KLHDC1	MST025	NM_172193	0.41	0.04	0.1211
LOC440259		XM_496056	0.50	0.11	0.0519
PTPRO v4	GLEPP1, PTP-U2, PTPU2	NM_030668	0.44	0.10	0.1133
SNF1LK	MSK, SIK	NM_173354	0.25	0.04	0.9000
STAT5A	MGF, STAT5	NM_003152	0.45	0.05	0.0696
K-ALPHA-1	α-tubulin	NM_006082	0.26	0.17	1.0000
