# Supplementary material for: Negative Regulators of Insulin Signaling Revealed in a Genome-Wide Functional Screen
Source: PLoS One. 2009 Sep 3;4(9):e6871. doi: 10.1371/journal.pone.0006871 (PMC2731165; doi:10.1371/journal.pone.0006871)
Supplement: Table S3 — Phospho-(Ser473)/Total AKT Multiplex Immunoassay Data. Shown here are the raw pAKT and tAKT data sets as well as the calculated pAKT/tAKT ratios for each of the selected screen hits (listed in alphabetical order) that were assayed as detailed in the Methods. The four independent experiments that were performed are color-coded with additional specific time-course differences indicated in the table heading. The “0” time-point corresponds to no insulin treatment while the insulin (50 nM) treatment times are indicated in minutes (m) and hours (h). Generally, for each time- point examined two or three replicates were performed per experiment and up to three independent experiments were carried out for each assayed cDNA. For comparative purposes, the computed average pAKT/tAKT ratios ± the standard deviations for the indicated time-points from each independent experiment performed on an individual cDNA are plotted in Figure S2 relative to the corresponding microplate values obtained with the negative vector control, pCMV6-XL5, and the positive control, Grb10. (0.05 MB PDF) [file pone.0006871.s005.pdf]

Table S3. Phospho-(Ser473)/Total AKT Multiplex Immunoassay Data. Shown here are the raw pAKT and tAKT data sets as well as the calculated pAKT/tAKT ratios for each of the selected screen hits (listed in alphabetical order) that were assayed as detailed in the Methods. The four independent experiments that were performed are color-coded with additional specific time-course differences indicated in the table heading. The "0" time-point corresponds to no insulin treatment while the insulin (50 nM) treatment times are indicated in minutes (m) and hours (h). Generally, for each time- point examined two or three replicates were performed per experiment and up to three independent experiments were carried out for each assayed cDNA. For comparative purposes, the computed average pAKT/tAKT ratios ± the standard deviations for the indicated time-points from each independent experiment performed on an individual cDNA are plotted in Figure S2 relative to the corresponding microplate values obtained with the negative vector control, pCMV6-XL5, and the positive control, Grb10.

Table S3

				raw data (pAkt)						raw data (tAkt)						raw data (pAkt/tAkt)						Average (pAkt/tAkt)						Stdev (pAkt/tAkt)					
cDNA	Experiment1	plate#	replicate#	0	5m	30m	1.5h	3h	6h	0	5m	30m	1.5h	3h	6h	0	5m	30m	1.5h	3h	6h	0	5m	30m	1.5h	3h	6h	0	5m	30m	1.5h	3h	6h
cDNA	Experiment2	plate#	replicate#	0	25m	2.5h	8.5h	16.5h	24.5h	0	25m	2.5h	8.5h	16.5h	24.5h	0	25m	2.5h	8.5h	16.5h	24.5h	0	25m	2.5h	8.5h	16.5h	24.5h	0	25m	2.5h	8.5h	16.5h	24.5h
cDNA	Experiment3	plate#	replicate#	0	40m	3.5h	9h	17.5h	25h	0	40m	3.5h	9h	17.5h	25h	0	40m	3.5h	9h	17.5h	25h	0	40m	3.5h	9h	17.5h	25h	0	40m	3.5h	9h	17.5h	25h
cDNA	Experiment4	plate#	replicate#	0	40m	2.5h	8h	17h	24h	0	40m	2.5h	8h	17h	24h	0	40m	2.5h	8h	17h	24h	0	40m	2.5h	8h	17h	24h	0	40m	2.5h	8h	17h	24h
ABR v1	Experiment2	p4	r1	954	8460	7244	2275	2571	3599	22305	19238	20156	22461	23381	22733	0.04	0.44	0.36	0.10	0.11	0.16	0.04	0.52	0.40	0.12	0.12	0.19	0.00	0.07	0.05	0.02	0.03	0.06
ABR v1	Experiment2	p4	r2	929	11742	9644	3413	3572	5889	23619	20303	20905	24920	22468	23243	0.04	0.58	0.46	0.14	0.16	0.25												
ABR v1	Experiment2	p4	r3	938	10254	7020	2797	2458	3881	23095	18962	18541	23173	23185	25221	0.04	0.54	0.38	0.12	0.11	0.15												
ABR v1	Experiment4	p2	r1	324	3163	2980	2409	1248	2330	10565	8420	9323	10422	10529	9899	0.03	0.38	0.32	0.23	0.12	0.24	0.04	0.40	0.33	0.25	0.17	0.25	0.02	0.03	0.02	0.04	0.04	0.02
ABR v1	Experiment4	p2	r2	231	3329	2980	3021	1934	2814	10718	8564	9172	10384	10305	10220	0.02	0.39	0.32	0.29	0.19	0.28												
ABR v1	Experiment4	p2	r3	652	4171	3100	2139	1763	2397	10566	9627	8621	9509	9232	9602	0.06	0.43	0.36	0.22	0.19	0.25												
BCL2L1 v1	Experiment3	p1	r1	2592	14188	13110	10465	5013	7805	24779	17947	19368	21714	17281	16680	0.10	0.79	0.68	0.48	0.29	0.47	0.10	0.82	0.64	0.52	0.29	0.42	0.00	0.04	0.05	0.06	0.00	0.07
BCL2L1 v1	Experiment3	p1	r2	2246	14047	10728	9684	5516	7201	21844	16565	17573	17155	18923	19805	0.10	0.85	0.61	0.56	0.29	0.36												
C7orf27	Experiment2	p5	r1	2016	12105	10515	8489	4499	6253	19378	17125	18548	18286	18898	18324	0.10	0.71	0.57	0.46	0.24	0.34	0.08	0.69	0.59	0.45	0.24	0.33	0.02	0.02	0.05	0.04	0.01	0.03
C7orf27	Experiment2	p5	r2	1263	13281	12879	9054	4735	7003	22214	19276	19900	18810	19312	19460	0.06	0.69	0.65	0.48	0.25	0.36												
C7orf27	Experiment2	p5	r3	1917	11569	9617	7266	4655	5983	21270	17215	17041	17830	20630	20008	0.09	0.67	0.56	0.41	0.23	0.30												
CREB1 vB	Experiment4	p1	r1	422	4099	3936	3583	1936	3276	9374	8465	8804	8896	9058	9104	0.05	0.48	0.45	0.40	0.21	0.36	0.06	0.48	0.43	0.37	0.23	0.32	0.02	0.04	0.03	0.08	0.02	0.05
CREB1 vB	Experiment4	p1	r2	392	4143	3885	3777	2333	3075	9552	7973	8560	8833	9147	8996	0.04	0.52	0.45	0.43	0.26	0.34												
CREB1 vB	Experiment4	p1	r3	820	3830	3203	2274	2073	2472	9788	8732	8121	8208	9751	9338	0.08	0.44	0.39	0.28	0.21	0.26												
CRTC2	Experiment4	p1	r1	780	2634	2494	1659	993	1682	8203	6903	7977	8672	8704	8335	0.10	0.38	0.31	0.19	0.11	0.20	0.09	0.37	0.34	0.20	0.12	0.21	0.01	0.03	0.06	0.02	0.01	0.03
CRTC2	Experiment4	p1	r2	746	2852	3137	1867	1198	2052	8537	7267	7711	8370	9001	8632	0.09	0.39	0.41	0.22	0.13	0.24												
CRTC2	Experiment4	p1	r3	689	2585	1964	1463	1064	1665	8294	7635	6855	8189	8649	9056	0.08	0.34	0.29	0.18	0.12	0.18												
DOK2 v1	Experiment2	p10	r1	2194	8469	9185	4840	4047	5287	23123	22511	23402	22500	23814	23455	0.09	0.38	0.39	0.22	0.17	0.23	0.08	0.39	0.40	0.19	0.15	0.19	0.02	0.05	0.05	0.02	0.02	0.04
DOK2 v1	Experiment2	p10	r2	1444	9872	9916	4340	3404	4589	23622	22081	22123	23997	24213	22426	0.06	0.45	0.45	0.18	0.14	0.20												
DOK2 v1	Experiment2	p10	r3	1985	7819	6996	3652	3218	3392	23732	21974	20276	21553	22571	23857	0.08	0.36	0.35	0.17	0.14	0.14												
DUSP1	Experiment1	p3	r1	2122	12070	13313	12648	12490	12520	24993	22131	21708	20505	20977	21218	0.08	0.55	0.61	0.62	0.60	0.59	0.08	0.51	0.62	0.56	0.51	0.50	0.01	0.04	0.08	0.09	0.07	0.08
DUSP1	Experiment1	p3	r2	2182	11278	12484	9949	10175	9726	26516	24100	22985	21741	22237	22384	0.08	0.47	0.54	0.46	0.46	0.43												
DUSP1	Experiment1	p3	r3	1540	12070	15428	14122	11333	11958	25631	23679	22230	23471	23227	24564	0.06	0.51	0.69	0.60	0.49	0.49												
DUSP1	Experiment2	p12	r1	3064	14384	14750	10585	6727	9047	26185	21341	23150	22240	26052	25404	0.12	0.67	0.64	0.48	0.26	0.36	0.10	0.67	0.67	0.46	0.27	0.39	0.03	0.01	0.04	0.05	0.04	0.05
DUSP1	Experiment2	p12	r2	1910	15597	16485	10433	6662	9700	28330	23751	23257	25811	28179	26184	0.07	0.66	0.71	0.40	0.24	0.37												
DUSP1	Experiment2	p12	r3	2825	14985	14145	12299	8425	11735	26824	22362	21322	24646	26638	25805	0.11	0.67	0.66	0.50	0.32	0.45												
DUSP10 v1	Experiment2	p11	r1	4797	14444	12604	10329	8785	10514	25224	24068	23700	25495	26923	26162	0.19	0.60	0.53	0.41	0.33	0.40	0.16	0.66	0.58	0.37	0.30	0.36	0.05	0.05	0.04	0.04	0.06	0.04
DUSP10 v1	Experiment2	p11	r2	2478	14439	14149	8586	6404	8394	25848	21837	23767	26198	27929	26369	0.10	0.66	0.60	0.33	0.23	0.32												
DUSP10 v1	Experiment2	p11	r3	4747	15424	12649	8551	7883	9697	26274	21859	20839	22808	22822	26969	0.18	0.71	0.61	0.37	0.35	0.36												
DUSP4 v1	Experiment1	p3	r1	1685	10872	10548	8465	9209	8498	22375	21334	19179	18432	18169	18645	0.08	0.51	0.55	0.46	0.51	0.46	0.07	0.47	0.60	0.50	0.49	0.46	0.02	0.03	0.05	0.04	0.03	0.01
DUSP4 v1	Experiment1	p3	r2	2209	11421	11256	9383	9879	9647	24263	24423	19260	19053	19420	20375	0.09	0.47	0.58	0.49	0.51	0.47												
DUSP4 v1	Experiment1	p3	r3	1316	9945	13485	11272	10039	10584	26329	22481	20614	21032	22294	23136	0.05	0.44	0.65	0.54	0.45	0.46												
DUSP4 v1	Experiment2	p11	r1	3658	15342	15958	9065	8820	10489	28118	23836	23519	26174	28914	26874	0.13	0.64	0.68	0.35	0.31	0.39	0.11	0.71	0.71	0.38	0.29	0.39	0.05	0.08	0.04	0.03	0.04	0.01
DUSP4 v1	Experiment2	p11	r2	1550	17863	18044	10497	6373	10536	27277	22216	23684	27490	26345	25914	0.06	0.80	0.76	0.38	0.24	0.41												
DUSP4 v1	Experiment2	p11	r3	3853	15959	15742	10464	8883	9571	25839	23481	22467	26010	27711	25338	0.15	0.68	0.70	0.40	0.32	0.38												
DUSP6 v1	Experiment1	p2	r1	1950	12307	15626	12754	10470	10531	29190	26140	24555	25166	24961	25900	0.07	0.47	0.64	0.51	0.42	0.41	0.06	0.50	0.65	0.54	0.47	0.41	0.01	0.03	0.05	0.05	0.07	0.05
DUSP6 v1	Experiment1	p2	r2	1460	12741	14927	13069	13098	11858	27912	23919	21248	21936	24096	25508	0.05	0.53	0.70	0.60	0.54	0.46												
DUSP6 v1	Experiment1	p2	r3	2030	12820	13917	12882	11513	11141	31146	26006	23101	24817	26394	29838	0.07	0.49	0.60	0.52	0.44	0.37												
DUSP6 v1	Experiment2	p11	r1	2944	11386	11701	6393	6068	8597	25236	22329	24564	26357	26806	29248	0.12	0.51	0.48	0.24	0.23	0.29	0.11	0.58	0.53	0.23	0.22	0.29	0.02	0.08	0.06	0.01	0.03	0.03
DUSP6 v1	Experiment2	p11	r2	2292	14331	14126	6063	5079	6722	25323	21445	23821	27886	27510	26009	0.09	0.67	0.59	0.22	0.18	0.26												
DUSP6 v1	Experiment2	p11	r3	3279	11882	10621	5568	6261	8021	25724	21143	20218	24221	25152	25052	0.13	0.56	0.53	0.23	0.25	0.32												
DUSP7	Experiment1	p2	r1	2629	12490	17434	14840	12785	13792	30149	27594	26794	27018	26404	26371	0.09	0.45	0.65	0.55	0.48	0.52	0.08	0.51	0.71	0.62	0.55	0.54	0.01	0.06	0.05	0.07	0.07	0.07
DUSP7	Experiment1	p2	r2	2007	14285	16700	15471	14773	15389	26763	24690	22357	22304	24038	24603	0.07	0.58	0.75	0.69	0.61	0.63												
DUSP7	Experiment1	p2	r3	2349	13884	17996	15882	14341	13753	30208	27405	24364	25445	26592	28644	0.08	0.51	0.74	0.62	0.54	0.48												
DUSP7	Experiment2	p11	r1	3095	17048	14138	9377	7778	8748	24647	22097	21225	24953	28862	25725	0.13	0.77	0.67	0.38	0.27	0.34	0.11	0.74	0.75	0.39	0.28	0.33	0.03	0.03	0.11	0.02	0.01	0.07
DUSP7	Experiment2	p11	r2	1773	15103	19150	10987	8030	10360	27133	21296	21890	26916	28215	25843	0.07	0.71	0.87	0.41	0.28	0.40												
DUSP7	Experiment2	p11	r3	3397	15918	15443	9776	7746	4830	27335	21427	21841	25071	27199	19178	0.12	0.74	0.71	0.39	0.28	0.25												
DUSP10 v1	Experiment1	p2	r1	2388	12524	15390	12315	11186	11701	30456	26922	24788	25224	24725	25638	0.08	0.47	0.62	0.49	0.45	0.46	0.08	0.51	0.68	0.55	0.50	0.55	0.00	0.05	0.08	0.07	0.07	0.15
DUSP10 v1	Experiment1	p2	r2	2373	13013	17305	13394	13747	16383	30053	23399	22511	21341	23759	22945	0.08	0.56	0.77	0.63	0.58	0.71												
DUSP10 v1	Experiment1	p2	r3	2401	12550	15885	13239	11541	11957	29300	24175	24626	25332	24059	25473	0.08	0.52	0.65	0.52	0.48	0.47												
FLJ21438	Experiment1	p6	r1	2630	13412	13338	12633	11313	9194	17961	16546	16263	16344	17319	17784	0.15	0.81	0.82	0.77	0.65	0.52	0.15	0.83	0.92	0.78	0.72	0.77	0.01	0.05	0.11	0.11	0.10	0.29
FLJ21438	Experiment1	p6	r2	2490	14029	14888	12246	13588	12278	16071	15727	14433	13663	16349	17069	0.15	0.89	1.03	0.90	0.83	0.72												
FLJ21438	Experiment1	p6	r3	3342	13884	16306	12550	13600	13251	21021	17473	18007	18708	20102	12235	0.16	0.79	0.91	0.67	0.68	1.08												
FLJ21438	Experiment2	p7	r1	2281	15662	16234	9478	5759	7345	23098	18529	20482	19550	23135	21434	0.10	0.85	0.79	0.48	0.25	0.34	0.10	0.95	0.92	0.55	0.30	0.39	0.01	0.14	0.19	0.08	0.06	0.06
FLJ21438	Experiment2	p7	r2	2140	21499	23466	13483	8113	9400	23775	19486	20594	21238	22552	20666	0.09	1.10	1.14	0.63	0.36	0.45												
FLJ21438	Experiment2	p7	r3	2587	17132	15621	11070	6451	7284	22498	19110	18707	20678	22942	20330	0.11	0.90	0.84	0.54	0.28	0.36												
FLJ22318	Experiment2	p7	r1	3605	13126	13077	10034	7180	8245	21375	19052	19424	19628	22137	19197	0.17	0.69	0.67	0.51	0.32	0.43	0.15	0.69	0.70	0.50	0.29	0.34	0.05	0.06	0.04	0.05	0.05	0.08
FLJ22318	Experiment2	p7	r2	2120	14351	15285	9485	5427	6216	22656	18840	20650	21568	22509	21741	0.09	0.76	0.74	0.44	0.24	0.29												
FLJ22318	Experiment2	p7	r3	3933	12250	12054	10384	6252	5741	21154	19313	17670	19241	19575	19456	0.19	0.63	0.68	0.54	0.32	0.30												
FOXO3A v1	Experiment4	p1	r1	2050	8624	8096	6821	4006	6290	10902	9878	10025	9601	10081	9125	0.19	0.87	0.81	0.71	0.40	0.69	0.16	0.78	0.69	0.60	0.35	0.52	0.05	0.13	0.11	0.13	0.06	0.16
FOXO3A v1	Experiment4	p1	r2	1051	5754	5598	4801	2973	3976	10357	9021	9541	10640	10799	10776	0.10	0.64	0.59	0.45	0.28	0.37												
FOXO3A v1	Experiment4	p1	r3	1998	8614	6736	5825	3632	4772	11046	10211	10028	9158	9810	9649	0.18	0.84	0.67	0.64	0.37	0.49												
Grb10	Experiment1	p1	r1	1468	5204	5545	4748	5310	4949	19091	18134	17083	17096	19631	19441	0.08	0.29	0.32	0.28	0.27	0.25												
Grb10	Experiment1	p2	r1	1820	6733	7467	6761	6073	5203	28587	25637	25358	26408	26013	28606	0.06	0.26	0.29	0.26	0.23	0.18												
Grb10	Experiment1	p3	r1	1219	5741	5354	4421	4367	3534	24217	23913	22859	22655	23877	25472	0.05	0.24	0.23	0.20	0.18	0.14												
Grb10	Experiment1	p4	r1	1468	5204	5545	4748	5310	4949	19091	18134	17083	17096	19631	19441	0.08	0.29	0.32	0.28	0.27	0.25												
Grb10	Experiment1	p5	r1	1166	3010	3055	3264	3366	3290	15887	14907	15563	17118	19141	19959	0.07	0.20	0.20	0.19	0.18	0.16	0.08	0.23	0.22	0.23	0.19	0.17	0.00	0.04	0.03	0.05	0.03	0.01
Grb10	Experiment1	p5	r2	1429	4058	4183	4706	3917	3491	18062	15788	17392	17637	18298	19744	0.08	0.26	0.24	0.27	0.21	0.18												
Grb10	Experiment1	p6	r1	1814	4717	4760	4678	4393	4962	17374	14709	16333	16690	17590	19844	0.10	0.32	0.29	0.28	0.25	0.25	0.11	0.34	0.34	0.32	0.28	0.29	0.00	0.03	0.06	0.06	0.04	0.06
Grb10	Experiment1	p6	r2	1895	5846	6011	6109	5199	6181	17302	15993	15771	16795	17206	18823	0.11	0.37	0.38	0.36	0.30	0.33												
Grb10	Experiment1	p7	r1	2788	6897	7043	6353	6888	5860	23488	21097	20632	20325	22226	23458	0.12	0.33	0.34	0.31	0.31	0.25	0.12	0.42	0.40	0.38	0.34	0.30	0.00	0.13	0.08	0.10	0.04	0.07
Grb10	Experiment1	p7	r2	3160	12103	10426	10359	8417	8362	25722	23967	23073	23031	23252	24193	0.12	0.50	0.45	0.45	0.36	0.35												
Grb10	Experiment2	p1	r1	1153	5654	4616	1468	1295	1577	15798	15181	15018	15862	16396	15543	0.07	0.37	0.31	0.09	0.08	0.10	0.08	0.52	0.34	0.09	0.07	0.10	0.02	0.21	0.04	0.00	0.01	0.00
Grb10	Experiment2	p1	r2	1471	9997	5973	1488	1107	1788	15488	14996	16467	15792	16512	16629	0.09	0.67	0.36	0.09	0.07	0.11												
Grb10	Experiment2	p2	r1	1494	6912	5547	1909	1346	1584	21906	20261	20281	22069	22730	22005	0.07	0.34	0.27	0.09	0.06	0.07	0.08	0.41	0.33	0.09	0.06	0.08	0.02	0.10	0.09	0.01	0.00	0.01
Grb10	Experiment2	p2	r2	1945	10003	8582	2167	1443	2011	21504	20991	21774	22403	22190	22455	0.09	0.48	0.39	0.10	0.07	0.09												
Grb10	Experiment2	p3	r1	1078	6345	5636	1736	1001	1462	21801	21059	21857	22162	22482	23935	0.05	0.30	0.26	0.08	0.04	0.06	0.05	0.39	0.28	0.08	0.05	0.07	0.01	0.13	0.03	0.00	0.01	0.01
Grb10	Experiment2	p3	r2	1400	10446	6196	1668	1389	1753	23398	21456	20996	21328	23803	23512	0.06	0.49	0.30	0.08	0.06	0.07												
Grb10	Experiment2	p4	r1	1579	7836	5641	1784	1317	1902	25610	23769	23270	24100	26188	27884	0.06	0.33	0.24	0.07	0.05	0.07	0.06	0.44	0.28	0.11	0.06	0.12	0.00	0.16	0.06	0.05	0.01	0.07
Grb10	Experiment2	p4	r2	1725	12805	7763	3386	1974	4251	25340	23208	23803	24526	28658	25097	0.07	0.55	0.33	0.14	0.07	0.17												
Grb10	Experiment2	p5	r1	1442	6840	6655	1930	1390	1465	21441	20297	20911	21099	22699	22370	0.07	0.34	0.32	0.09	0.06	0.07	0.07	0.41	0.42	0.11	0.07	0.09	0.01	0.11	0.15	0.03	0.01	0.03
Grb10	Experiment2	p5	r2	1773	9943	10970	2688	1683	2276	21742	20360	20812	21132	22059	21680	0.08	0.49	0.53	0.13	0.08	0.10												
Grb10	Experiment2	p6	r1	1977	7265	6796	2284	1460	2092	21843	21599	20122	21030	18369	24795	0.09	0.34	0.34	0.11	0.08	0.08	0.08	0.38	0.37	0.11	0.07	0.09	0.01	0.07	0.04	0.01	0.01	0.01
Grb10	Experiment2	p6	r2	1762	9249	8051	2514	1448	2233	22740	21499	20338	21602	20784	22466	0.08	0.43	0.40	0.12	0.07	0.10												
Grb10	Experiment2	p7	r1	1725	8096	7939	2447	1594	2338	21973	20842	20201	22709	24234	23748	0.08	0.39	0.39	0.11	0.07	0.10	0.08	0.42	0.44	0.13	0.07	0.12	0.00	0.05	0.07	0.03	0.01	0.03
Grb10	Experiment2	p7	r2	1921	9635	10810	3417	2181	3648	23068	20999	22283	23182	26223	25374	0.08	0.46	0.49	0.15	0.08	0.14												
Grb10	Experiment2	p8	r1	1755	8476	8022	2635	1562	2309	23661	22642	22319	23512	23831	24799	0.07	0.37	0.36	0.11	0.07	0.09	0.09	0.40	0.42	0.13	0.07	0.10	0.02	0.03	0.09	0.02	0.01	0.01
Grb10	Experiment2	p8	r2	2727	9599	12088	3547	1943	2934	27248	23020	24660	25450	26248	26178	0.10	0.42	0.49	0.14	0.07	0.11												
Grb10	Experiment2	p9	r1	1986	9015	9555	2930	1955	2355	28679	26784	26444	27802	29444	28858	0.07	0.34	0.36	0.11	0.07	0.08	0.08	0.39	0.41	0.12	0.07	0.09	0.02	0.08	0.07	0.02	0.00	0.01
Grb10	Experiment2	p9	r2	2561	12193	11763	3856	2039	2999	28175	27069	25814	29691	30423	29573	0.09	0.45	0.46	0.13	0.07	0.10												
Grb10	Experiment2	p10	r1	1833	7544	6310	1888	1271	1402	23102	21951	21287	21382	22090	23632	0.08	0.34	0.30	0.09	0.06	0.06	0.08	0.40	0.39	0.09	0.06	0.07	0.00	0.08	0.14	0.01	0.00	0.01
Grb10	Experiment2	p10	r2	2150	10198	10587	2463	1452	1934	25113	22156	21478	24339	24917	25173	0.09	0.46	0.49	0.10	0.06	0.08												
Grb10	Experiment2	p11	r1	1808	8249	7884	2329	1489	2137	25762	23862	23274	24358	25167	26419	0.07	0.35	0.34	0.10	0.06	0.08	0.07	0.38	0.39	0.11	0.06	0.09	0.00	0.05	0.08	0.02	0.01	0.01
Grb10	Experiment2	p11	r2	2044	9427	10094	2909	1758	2449	26551	22651	22535	24394	26517	25713	0.08	0.42	0.45	0.12	0.07	0.10												
Grb10	Experiment2	p12	r1	1759	7138	7183	2340	1739	2153	24984	22017	22425	23862	24207	23905	0.07	0.32	0.32	0.10	0.07	0.09	0.08	0.41	0.44	0.11	0.08	0.10	0.02	0.12	0.17	0.02	0.01	0.02
Grb10	Experiment2	p12	r2	2144	11082	12473	2858	2040	2772	22932	22516	22154	23372	25401	23674	0.09	0.49	0.56	0.12	0.08	0.12												

Table S3

				raw data (pAkt)						raw data (tAkt)						raw data (pAkt/tAkt)						Average (pAkt/tAkt)						Stdev (pAkt/tAkt)					
cDNA	Experiment1	plate#	replicate#	0	5m	30m	1.5h	3h	6h	0	5m	30m	1.5h	3h	6h	0	5m	30m	1.5h	3h	6h	0	5m	30m	1.5h	3h	6h	0	5m	30m	1.5h	3h	6h
cDNA	Experiment2	plate#	replicate#	0	25m	2.5h	8.5h	16.5h	24.5h	0	25m	2.5h	8.5h	16.5h	24.5h	0	25m	2.5h	8.5h	16.5h	24.5h	0	25m	2.5h	8.5h	16.5h	24.5h	0	25m	2.5h	8.5h	16.5h	24.5h
cDNA	Experiment3	plate#	replicate#	0	40m	3.5h	9h	17.5h	25h	0	40m	3.5h	9h	17.5h	25h	0	40m	3.5h	9h	17.5h	25h	0	40m	3.5h	9h	17.5h	25h	0	40m	3.5h	9h	17.5h	25h
cDNA	Experiment4	plate#	replicate#	0	40m	2.5h	8h	17h	24h	0	40m	2.5h	8h	17h	24h	0	40m	2.5h	8h	17h	24h	0	40m	2.5h	8h	17h	24h	0	40m	2.5h	8h	17h	24h
Grb10	Experiment2	p13	r1	2403	10737	10738	2966	1757	2739	27177	26811	26117	27510	26799	29021	0.09	0.40	0.41	0.11	0.07	0.09	0.10	0.51	0.51	0.14	0.08	0.12	0.01	0.15	0.13	0.04	0.01	0.03
Grb10	Experiment2	p13	r2	2872	15151	13725	4597	2489	3792	27292	24603	22807	26900	28749	26612	0.11	0.62	0.60	0.17	0.09	0.14												
Grb10	Experiment3	p1	r1	1600	5751	4329	2143	1330	1711	13477	15110	16871	16464	13623	15198	0.12	0.38	0.26	0.13	0.10	0.11	0.12	0.41	0.31	0.17	0.11	0.14	0.01	0.04	0.07	0.05	0.02	0.03
Grb10	Experiment3	p1	r2	1960	7145	5555	3266	1657	2684	15202	16452	15515	15788	12700	16736	0.13	0.43	0.36	0.21	0.13	0.16												
Grb10	Experiment4	p1	r1	732	2401	2126	1276	774	1189	9003	9168	8331	8933	10095	9705	0.08	0.26	0.26	0.14	0.08	0.12	0.08	0.30	0.25	0.15	0.09	0.13	0.00	0.05	0.00	0.01	0.01	0.02
Grb10	Experiment4	p1	r2	793	3161	2084	1438	942	1328	10190	9391	8214	9071	9647	9228	0.08	0.34	0.25	0.16	0.10	0.14												
Grb10	Experiment4	p2	r1	986	3438	3608	2269	1331	1942	10443	10577	10689	10699	11288	10871	0.09	0.33	0.34	0.21	0.12	0.18	0.10	0.37	0.38	0.22	0.12	0.18	0.00	0.07	0.06	0.01	0.00	0.00
Grb10	Experiment4	p2	r2	1094	4660	4676	2612	1310	1975	11099	11036	11054	11261	11493	10862	0.10	0.42	0.42	0.23	0.11	0.18												
Grb10	Experiment4	p3	r1	1193	4107	3513	2107	1153	1695	10630	10701	10036	10347	10758	10911	0.11	0.38	0.35	0.20	0.11	0.16	0.11	0.41	0.41	0.22	0.11	0.18	0.01	0.04	0.09	0.02	0.00	0.04
Grb10	Experiment4	p3	r2	1095	4313	4742	2187	1160	2055	10837	9807	9985	9472	10236	9823	0.10	0.44	0.47	0.23	0.11	0.21												
KIAA0672	Experiment2	p8	r1	2039	11552	11814	8195	5360	7925	22312	21014	21617	24406	21490	25783	0.09	0.55	0.55	0.34	0.25	0.31	0.08	0.56	0.62	0.32	0.23	0.33	0.01	0.01	0.07	0.03	0.02	0.04
KIAA0672	Experiment2	p8	r2	1580	12827	15448	6923	6044	8342	21914	22153	23157	24478	24802	22069	0.07	0.58	0.67	0.28	0.24	0.38												
KIAA0672	Experiment2	p8	r3	1817	11714	13560	6876	5154	7173	22248	20892	20882	20449	24675	23555	0.08	0.56	0.65	0.34	0.21	0.30												
KIAA0980	Experiment2	p8	r1	2289	12045	13220	8263	5653	7260	23064	20360	20636	24051	24330	24127	0.10	0.59	0.64	0.34	0.23	0.30	0.10	0.67	0.68	0.39	0.24	0.34	0.01	0.07	0.12	0.05	0.04	0.04
KIAA0980	Experiment2	p8	r2	2515	16439	18573	11420	7388	10500	27438	22732	22961	25621	25800	27782	0.09	0.72	0.81	0.45	0.29	0.38												
KIAA0980	Experiment2	p8	r3	2535	13714	11320	8566	4995	7733	22509	19654	19267	22980	23185	23270	0.11	0.70	0.59	0.37	0.22	0.33												
KIAA1102	Experiment1	p1	r1	1529	9456	12528	9420	8881	11198	16615	14625	16161	15023	15955	18363	0.09	0.65	0.78	0.63	0.56	0.61	0.10	0.61	0.75	0.61	0.58	0.62	0.01	0.05	0.08	0.02	0.04	0.05
KIAA1102	Experiment1	p1	r2	1809	8644	10326	8707	9110	9675	16833	15368	15558	14941	16059	16704	0.11	0.56	0.66	0.58	0.57	0.58												
KIAA1102	Experiment1	p1	r3	1777	9841	11031	8945	10118	11300	17957	15502	13661	14644	16037	16785	0.10	0.63	0.81	0.61	0.63	0.67												
KIAA1102	Experiment2	p10	r1	1605	5449	9382	4494	4780	7096	20698	17704	18731	21174	18485	19688	0.08	0.31	0.50	0.21	0.26	0.36	0.07	0.41	0.52	0.20	0.23	0.33	0.01	0.09	0.09	0.01	0.04	0.05
KIAA1102	Experiment2	p10	r2	1467	8134	11261	4241	5296	6932	20683	17887	18246	21155	21348	19672	0.07	0.45	0.62	0.20	0.25	0.35												
KIAA1102	Experiment2	p10	r3	1363	8605	7793	3643	3813	5340	22039	18363	17836	19747	20479	19639	0.06	0.47	0.44	0.18	0.19	0.27												
KIAA1102	Experiment4	p3	r1	1411	4031	4936	5156	3884	5185	9890	8635	9308	9764	10707	9992	0.14	0.47	0.53	0.53	0.36	0.52	0.14	0.53	0.56	0.55	0.36	0.53	0.01	0.06	0.06	0.03	0.00	0.02
KIAA1102	Experiment4	p3	r2	1384	4947	5494	5481	3679	5499	10632	8919	8747	9444	10109	10570	0.13	0.55	0.63	0.58	0.36	0.52												
KIAA1102	Experiment4	p3	r3	1433	5138	4566	5047	3468	5114	10343	8930	8803	9393	9736	9096	0.14	0.58	0.52	0.54	0.36	0.56												
KLHDC1	Experiment2	p6	r1	3730	11626	10513	9359	7760	8862	20488	18176	18510	17959	20836	20726	0.18	0.64	0.57	0.52	0.37	0.43	0.16	0.66	0.61	0.50	0.34	0.38	0.02	0.03	0.09	0.03	0.06	0.05
KLHDC1	Experiment2	p6	r2	2946	13654	14102	9238	6096	7205	21493	19647	19706	19588	22491	20799	0.14	0.69	0.72	0.47	0.27	0.35												
KLHDC1	Experiment2	p6	r3	3614	12941	10429	10281	7324	7663	21730	20195	19184	19855	19674	21817	0.17	0.64	0.54	0.52	0.37	0.35												
LOC440259	Experiment1	p6	r1	3310	14982	13470	12968	12373	12376	19071	17623	16677	18263	18945	19996	0.17	0.85	0.81	0.71	0.65	0.62	0.16	0.80	0.84	0.75	0.69	0.66	0.02	0.05	0.03	0.05	0.04	0.03
LOC440259	Experiment1	p6	r2	2346	12829	14014	12542	12702	11735	17684	16478	16345	15667	17563	17355	0.13	0.78	0.86	0.80	0.72	0.68												
LOC440259	Experiment1	p6	r3	3228	14107	15143	14116	13474	13492	19896	18424	17658	19315	19363	19786	0.16	0.77	0.86	0.73	0.70	0.68												
LOC440259	Experiment2	p8	r1	2334	14525	15331	10891	5659	8332	23869	22290	24079	23433	24484	24561	0.10	0.65	0.64	0.46	0.23	0.34	0.10	0.71	0.69	0.45	0.25	0.36	0.02	0.06	0.04	0.02	0.02	0.02
LOC440259	Experiment2	p8	r2	1996	17897	16697	10786	7068	9443	26579	23497	23869	25346	27480	26458	0.08	0.76	0.70	0.43	0.26	0.36												
LOC440259	Experiment2	p8	r3	3037	15977	15427	10692	6308	8545	24380	22313	21330	23029	24185	22998	0.12	0.72	0.72	0.46	0.26	0.37												
MAP2K2	Experiment1	p7	r1	3545	14328	14054	11772	10272	11073	19790	20086	18408	17905	17480	18971	0.18	0.71	0.76	0.66	0.59	0.58	0.16	0.78	0.76	0.67	0.61	0.59	0.01	0.16	0.03	0.02	0.03	0.01
MAP2K2	Experiment1	p7	r2	3499	18550	13542	11520	12662	12057	22168	19308	17386	16634	19624	20075	0.16	0.96	0.78	0.69	0.65	0.60												
MAP2K2	Experiment1	p7	r3	3684	14097	14897	13167	11110	11951	24025	21519	20441	20031	18694	20782	0.15	0.66	0.73	0.66	0.59	0.58												
MAP2K2	Experiment2	p4	r1	948	12252	10517	8685	4289	6875	22382	19419	18868	20579	21157	22882	0.04	0.63	0.56	0.42	0.20	0.30	0.05	0.69	0.62	0.44	0.24	0.32	0.01	0.07	0.08	0.04	0.03	0.03
MAP2K2	Experiment2	p4	r2	822	13217	12891	10672	6250	8140	24205	17096	18401	21780	23004	23219	0.03	0.77	0.70	0.49	0.27	0.35												
MAP2K2	Experiment2	p4	r3	1384	12531	10857	8079	5568	7057	23183	18565	18419	19900	23250	22935	0.06	0.67	0.59	0.41	0.24	0.31												
MAST3	Experiment1	p4	r1	2243	10373	10711	9507	10205	8610	18090	15480	13890	14046	15491	16781	0.12	0.67	0.77	0.68	0.66	0.51	0.11	0.67	0.73	0.68	0.63	0.55	0.02	0.03	0.06	0.01	0.04	0.04
MAST3	Experiment1	p4	r2	1628	10363	11068	9695	9297	9924	18424	14819	14328	14650	15882	17952	0.09	0.70	0.77	0.66	0.59	0.55												
MAST3	Experiment1	p4	r3	2500	10285	9776	10156	10551	10832	20273	15918	14827	14691	15981	18342	0.12	0.65	0.66	0.69	0.66	0.59												
MAST3	Experiment2	p1	r1	1360	11618	10991	5414	3215	5133	18780	15356	16284	17466	15266	14484	0.07	0.76	0.67	0.31	0.21	0.35	0.08	0.85	0.74	0.33	0.25	0.43	0.01	0.16	0.17	0.04	0.06	0.09
MAST3	Experiment2	p1	r2	1580	13403	11890	5671	4790	7629	16540	12935	12728	14952	15002	14397	0.10	1.04	0.93	0.38	0.32	0.53												
MAST3	Experiment2	p1	r3	1292	10306	8400	4374	3103	5638	18133	13671	13672	14420	14614	13893	0.07	0.75	0.61	0.30	0.21	0.41												
MAST4	Experiment1	p4	r1	4027	14702	18470	15703	16070	16657	22927	20480	19681	20035	21713	21237	0.18	0.72	0.94	0.78	0.74	0.78	0.17	0.74	0.94	0.83	0.76	0.80	0.03	0.02	0.06	0.08	0.03	0.12
MAST4	Experiment1	p4	r2	3198	15567	20129	15280	14861	13433	23897	20355	20114	19452	20145	19676	0.13	0.76	1.00	0.79	0.74	0.68												
MAST4	Experiment1	p4	r3	4574	15832	17851	18044	16120	19178	24406	21263	20067	19695	20425	20784	0.19	0.74	0.89	0.92	0.79	0.92												
MAST4	Experiment2	p1	r1	2174	14860	13782	9249	5085	7220	18721	15817	16553	15804	15152	15763	0.12	0.94	0.83	0.59	0.34	0.46	0.10	0.93	0.81	0.63	0.32	0.47	0.02	0.07	0.02	0.06	0.03	0.07
MAST4	Experiment2	p1	r2	1394	15456	12779	11168	5070	7691	16933	15598	15663	16124	15289	14155	0.08	0.99	0.82	0.69	0.33	0.54												
MAST4	Experiment2	p1	r3	1956	13260	11113	8724	4339	6220	17391	15436	14074	14242	15321	15399	0.11	0.86	0.79	0.61	0.28	0.40												
NCK2 v1	Experiment2	p10	r1	1286	8867	8122	2497	1770	2873	21798	20363	20046	22555	23931	22592	0.06	0.44	0.41	0.11	0.07	0.13	0.06	0.46	0.48	0.10	0.07	0.13	0.00	0.03	0.21	0.01	0.00	0.03
NCK2 v1	Experiment2	p10	r2	1296	10536	15231	2619	1837	3632	24254	21648	21374	24856	24629	23594	0.05	0.49	0.71	0.11	0.07	0.15												
NCK2 v1	Experiment2	p10	r3	1474	9141	5855	1984	1494	2197	24176	19790	18138	21588	22037	21332	0.06	0.46	0.32	0.09	0.07	0.10												
pCMV6-XL5	Experiment1	p1	r1	1940	9970	10337	8745	9307	10070	19779	18476	15344	16320	17697	17399	0.10	0.54	0.67	0.54	0.53	0.58												
pCMV6-XL5	Experiment1	p2	r1	2525	11558	13326	11052	11485	11869	27292	23653	22947	22962	23300	25012	0.09	0.49	0.58	0.48	0.49	0.47	0.10	0.55	0.66	0.51	0.50	0.50	0.01	0.09	0.12	0.03	0.01	0.03
pCMV6-XL5	Experiment1	p2	r2	2850	13935	15789	11325	12188	12760	26692	22630	21193	21381	24178	24525	0.11	0.62	0.75	0.53	0.50	0.52												
pCMV6-XL5	Experiment1	p3	r1	1683	9639	10772	9519	7864	8818	25499	21370	21576	21989	21253	22953	0.07	0.45	0.50	0.43	0.37	0.38	0.08	0.44	0.52	0.43	0.37	0.39	0.02	0.01	0.02	0.00	0.00	0.01
pCMV6-XL5	Experiment1	p3	r2	2604	10298	12095	10475	9322	10220	26746	23894	22657	24359	24831	25499	0.10	0.43	0.53	0.43	0.38	0.40												
pCMV6-XL5	Experiment1	p4	r1	1940	9970	10337	8745	9307	10070	19779	18476	15344	16320	17697	17399	0.10	0.54	0.67	0.54	0.53	0.58												
pCMV6-XL5	Experiment1	p5	r1	1695	6634	7931	7987	7588	7903	16293	14671	13780	15152	16812	17752	0.10	0.45	0.58	0.53	0.45	0.45	0.11	0.47	0.61	0.53	0.47	0.46	0.00	0.03	0.04	0.00	0.02	0.02
pCMV6-XL5	Experiment1	p5	r2	1851	7643	9081	8351	8803	9110	17217	15465	14227	15696	18116	19137	0.11	0.49	0.64	0.53	0.49	0.48												
pCMV6-XL5	Experiment1	p6	r1	3012	11293	11390	10994	10787	10860	17321	13707	14287	15453	15886	16770	0.17	0.82	0.80	0.71	0.68	0.65	0.16	0.85	0.82	0.75	0.69	0.67	0.02	0.04	0.03	0.05	0.02	0.03
pCMV6-XL5	Experiment1	p6	r2	2511	12102	11956	12341	10794	11815	17822	13826	14282	15846	15338	17109	0.14	0.88	0.84	0.78	0.70	0.69												
pCMV6-XL5	Experiment1	p7	r1	3785	15008	14280	12793	13285	13119	23930	18780	17195	18178	19757	20483	0.16	0.80	0.83	0.70	0.67	0.64	0.16	0.87	0.88	0.75	0.69	0.67	0.01	0.10	0.08	0.06	0.03	0.04
pCMV6-XL5	Experiment1	p7	r2	4111	17806	16877	14782	14646	15898	24779	18983	17998	18711	20539	22711	0.17	0.94	0.94	0.79	0.71	0.70												
pCMV6-XL5	Experiment2	p1	r1	1197	11097	9166	7791	4539	6597	17340	14568	14461	14492	15402	14503	0.07	0.76	0.63	0.54	0.29	0.45	0.07	0.81	0.65	0.52	0.30	0.48	0.01	0.07	0.02	0.02	0.00	0.03
pCMV6-XL5	Experiment2	p1	r2	1301	13124	9894	7658	4695	7568	16498	15146	14829	15228	15776	15196	0.08	0.87	0.67	0.50	0.30	0.50												
pCMV6-XL5	Experiment2	p2	r1	1613	13787	10304	9862	5419	7658	21968	18962	18804	19157	21550	20840	0.07	0.73	0.55	0.51	0.25	0.37	0.08	0.79	0.63	0.52	0.26	0.38	0.00	0.09	0.11	0.01	0.01	0.01
pCMV6-XL5	Experiment2	p2	r2	1939	17476	13682	10453	6084	8856	24863	20475	19373	19953	23169	22810	0.08	0.85	0.71	0.52	0.26	0.39												
pCMV6-XL5	Experiment2	p3	r1	1595	12289	11029	7810	4865	5991	24452	20228	19700	20660	22842	22290	0.07	0.61	0.56	0.38	0.21	0.27	0.06	0.57	0.59	0.39	0.23	0.29	0.00	0.05	0.04	0.02	0.02	0.04
pCMV6-XL5	Experiment2	p3	r2	1640	13410	12226	9393	5740	7364	26346	25031	19729	23466	24009	23087	0.06	0.54	0.62	0.40	0.24	0.32												
pCMV6-XL5	Experiment2	p4	r1	2234	15636	11863	11219	6289	7529	26008	20963	19673	23755	24878	23051	0.09	0.75	0.60	0.47	0.25	0.33	0.08	0.77	0.66	0.49	0.25	0.34	0.01	0.04	0.08	0.02	0.01	0.02
pCMV6-XL5	Experiment2	p4	r2	1902	18862	14952	11588	6326	9335	26262	23572	20826	23270	26575	26177	0.07	0.80	0.72	0.50	0.24	0.36												
pCMV6-XL5	Experiment2	p5	r1	1847	15016	10773	8845	4923	6213	21702	18423	19034	19308	21330	19505	0.09	0.82	0.57	0.46	0.23	0.32	0.09	0.80	0.66	0.49	0.24	0.35	0.00	0.01	0.14	0.04	0.01	0.04
pCMV6-XL5	Experiment2	p5	r2	2055	15168	15293	10117	5893	8165	23574	19105	20123	19727	23761	21837	0.09	0.79	0.76	0.51	0.25	0.37												
pCMV6-XL5	Experiment2	p6	r1	3609	14995	11712	9311	6627	8171	20896	18373	16846	18994	21448	22545	0.17	0.82	0.70	0.49	0.31	0.36	0.15	0.87	0.74	0.53	0.30	0.36	0.03	0.07	0.06	0.06	0.02	0.00
pCMV6-XL5	Experiment2	p6	r2	2708	18160	13134	10560	6694	8262	21497	19859	16840	18261	23275	22855	0.13	0.91	0.78	0.58	0.29	0.36												
pCMV6-XL5	Experiment2	p7	r1	2475	18189	16178	10096	6546	8775	22887	19970	19031	19545	23742	21625	0.11	0.91	0.85	0.52	0.28	0.41	0.10	0.91	0.85	0.50	0.28	0.42	0.01	0.00	0.00	0.02	0.00	0.01
pCMV6-XL5	Experiment2	p7	r2	2061	18686	17116	10441	7004	10573	22253	20608	19972	21303	25198	24763	0.09	0.91	0.86	0.49	0.28	0.43												
pCMV6-XL5	Experiment2	p8	r1	2504	16068	12936	9740	5430	7956	22839	19830	19480	20398	21941	21529	0.11	0.81	0.66	0.48	0.25	0.37	0.10	0.78	0.71	0.47	0.25	0.37	0.02	0.04	0.07	0.01	0.01	0.00
pCMV6-XL5	Experiment2	p8	r2	2211	16356	16383	11198	6688	8660	26100	21891	21583	24107	26221	23884	0.08	0.75	0.76	0.46	0.26	0.36												
pCMV6-XL5	Experiment2	p9	r1	2890	16339	15024	10972	6279	9862	28500	23549	24054	26053	27521	27575	0.10	0.69	0.62	0.42	0.23	0.36	0.09	0.68	0.61	0.43	0.24	0.35	0.01	0.01	0.02	0.01	0.01	0.01
pCMV6-XL5	Experiment2	p9	r2	2426	17864	14705	11988	7498	10671	30168	26457	24748	27340	30217	30604	0.08	0.68	0.59	0.44	0.25	0.35												
pCMV6-XL5	Experiment2	p10	r1	2493	13969	12339	7425	4204	4616	23374	21081	19568	19197	20859	19679	0.11	0.66	0.63	0.39	0.20	0.23	0.10	0.68	0.65	0.38	0.19	0.26	0.02	0.03	0.03	0.01	0.01	0.03
pCMV6-XL5	Experiment2	p10	r2	2060	15393	13074	7869	4135	5980	24618	22042	19375	21497	22710	21410	0.08	0.70	0.67	0.37	0.18	0.28												
pCMV6-XL5	Experiment2	p11	r1	4223	14927	12550	9956	6939	8462	27362	22798	21266	23565	26192	24615	0.15	0.65	0.59	0.42	0.26	0.34	0.12	0.70	0.69	0.45	0.28	0.37	0.05	0.06	0.14	0.04	0.02	0.03
pCMV6-XL5	Experiment2	p11	r2	2091	16269	15248	10464	7421	9192	26277	21966	19506	22044	25271	23703	0.08	0.74	0.78	0.47	0.29	0.39												
pCMV6-XL5	Experiment2	p12	r1	2949	14035	12343	9334	5568	8042	25182	20957	19388	19615	22151	20598	0.12	0.67	0.64	0.48	0.25	0.39	0.11	0.68	0.69	0.45	0.27	0.44	0.02	0.01	0.08	0.04	0.02	0.07
pCMV6-XL5	Experiment2	p12	r2	2259	13766	14766	9259	6631	10536	24291	20089	19600	22311	23382	21790	0.09	0.69	0.75	0.41	0.28	0.48												
pCMV6-XL5	Experiment2	p13	r1	4435	21855	17279	12740	7595	10901	27757	25023	24518	26080	28020	26923	0.16	0.87	0.70	0.49	0.27	0.40	0.13	0.80	0.79	0.55	0.30	0.43	0.04	0.11	0.12	0.09	0.04	0.04
pCMV6-XL5	Experiment2	p13	r2	2957	17069	19819	15872	8522	12099	28525	23697	22674	25737	26490	26416	0.10	0.72	0.87	0.62	0.32	0.46												
pCMV6-XL5	Experiment3	p1	r1	1657	9820	6660	7344	3957	5530	14811	12652	11136	13289	12774	13312	0.11	0.78	0.60	0.55	0.31	0.41	0.11	0.83	0.62	0.54	0.32	0.41	0.00	0.07	0.03	0.02	0.02	0.00
pCMV6-XL5	Experiment3	p1	r2	1586	11440	8396	7733	4895	6172	14047	12970	13084	14840	14413	15002	0.11	0.88	0.64	0.52	0.34	0.41												
pCMV6-XL5	Experiment4	p1	r1	759	3186	2962	2992	2313	2325	5595	7289	7637	8005	8319	8717	0.14	0.44	0.39	0.37	0.28	0.27	0.11	0.45	0.40	0.31	0.25	0.28	0.04	0.01	0.02	0.09	0.04	0.02
pCMV6-XL5	Experiment4	p1	r2	799	3527	3335	1933	1864	2566	9600	7767	8044	8045	8616	8681	0.08	0.45	0.41	0.24	0.22	0.30												

Table S3

				raw data (pAkt)						raw data (tAkt)						raw data (pAkt/tAkt)						Average (pAkt/tAkt)						Stdev (pAkt/tAkt)					
cDNA	Experiment1	plate#	replicate#	0	5m	30m	1.5h	3h	6h	0	5m	30m	1.5h	3h	6h	0	5m	30m	1.5h	3h	6h	0	5m	30m	1.5h	3h	6h	0	5m	30m	1.5h	3h	6h
cDNA	Experiment2	plate#	replicate#	0	25m	2.5h	8.5h	16.5h	24.5h	0	25m	2.5h	8.5h	16.5h	24.5h	0	25m	2.5h	8.5h	16.5h	24.5h	0	25m	2.5h	8.5h	16.5h	24.5h	0	25m	2.5h	8.5h	16.5h	24.5h
cDNA	Experiment3	plate#	replicate#	0	40m	3.5h	9h	17.5h	25h	0	40m	3.5h	9h	17.5h	25h	0	40m	3.5h	9h	17.5h	25h	0	40m	3.5h	9h	17.5h	25h	0	40m	3.5h	9h	17.5h	25h
cDNA	Experiment4	plate#	replicate#	0	40m	2.5h	8h	17h	24h	0	40m	2.5h	8h	17h	24h	0	40m	2.5h	8h	17h	24h	0	40m	2.5h	8h	17h	24h	0	40m	2.5h	8h	17h	24h
pCMV6-XL5	Experiment4	p2	r1	1327	5592	5034	4700	2695	3514	10755	8906	9283	9350	10113	9275	0.12	0.63	0.54	0.50	0.27	0.38	0.11	0.61	0.57	0.48	0.28	0.37	0.02	0.03	0.04	0.03	0.02	0.01
pCMV6-XL5	Experiment4	p2	r2	1148	5533	5878	4605	2926	3452	11584	9431	9821	10025	9958	9453	0.10	0.59	0.60	0.46	0.29	0.37												
pCMV6-XL5	Experiment4	p3	r1	1556	7018	5184	5050	3165	3768	10623	9641	9392	9167	9567	9222	0.15	0.73	0.55	0.55	0.33	0.41	0.13	0.71	0.58	0.52	0.33	0.40	0.03	0.03	0.04	0.05	0.00	0.01
pCMV6-XL5	Experiment4	p3	r2	1237	6251	5715	4394	3256	3919	11193	9131	9313	9125	10043	10059	0.11	0.68	0.61	0.48	0.32	0.39												
PALD	Experiment2	p9	r1	1931	7502	8505	3942	3383	5355	24335	20343	21256	22229	23897	22885	0.08	0.37	0.40	0.18	0.14	0.23	0.07	0.42	0.41	0.18	0.14	0.24	0.01	0.06	0.05	0.02	0.01	0.03
PALD	Experiment2	p9	r2	1725	10116	10020	4742	3250	5745	25276	20717	21315	23561	21984	20962	0.07	0.49	0.47	0.20	0.15	0.27												
PALD	Experiment2	p9	r3	1860	9263	7699	3919	3087	5118	25091	22667	20638	23008	25644	24478	0.07	0.41	0.37	0.17	0.12	0.21												
PALD	Experiment4	p2	r1	720	3316	2829	2422	1639	2280	9517	9357	8700	8482	9166	8379	0.08	0.35	0.33	0.29	0.18	0.27	0.08	0.37	0.36	0.32	0.20	0.32	0.00	0.03	0.06	0.07	0.03	0.06
PALD	Experiment4	p2	r2	796	3570	3617	3359	2272	3569	10387	8939	8487	8519	9525	9156	0.08	0.40	0.43	0.39	0.24	0.39												
PALD	Experiment4	p2	r3	709	3031	2854	2299	1776	2630	9595	8472	8815	8473	9510	8685	0.07	0.36	0.32	0.27	0.19	0.30												
PEA15	Experiment3	p1	r1	2125	13408	12193	8809	5736	7192	17661	14797	18661	16427	16735	18344	0.12	0.91	0.65	0.54	0.34	0.39	0.12	0.92	0.64	0.53	0.35	0.35	0.00	0.02	0.02	0.01	0.01	0.06
PEA15	Experiment3	p1	r2	1951	12751	7999	7506	5609	6010	16520	13619	12665	14272	16023	19495	0.12	0.94	0.63	0.53	0.35	0.31												
PIK3R1 v1	Experiment1	p7	r1	4817	18291	19761	16532	15345	16089	22859	20631	19638	19579	19850	21428	0.21	0.89	1.01	0.84	0.77	0.75	0.18	0.90	0.99	0.85	0.78	0.74	0.03	0.06	0.02	0.02	0.01	0.02
PIK3R1 v1	Experiment1	p7	r2	3966	22058	21060	18570	17811	16945	25309	22688	21066	21967	23154	22662	0.16	0.97	1.00	0.85	0.77	0.75												
PIK3R1 v1	Experiment1	p7	r3	4814	18936	20768	17456	17442	16307	26078	22324	21372	19980	22023	23082	0.18	0.85	0.97	0.87	0.79	0.71												
PIK3R1 v1	Experiment2	p12	r1	1059	8546	8623	1957	918	1508	24370	20708	21783	22604	24054	24322	0.04	0.41	0.40	0.09	0.04	0.06	0.05	0.44	0.40	0.08	0.05	0.07	0.02	0.03	0.02	0.01	0.02	0.02
PIK3R1 v1	Experiment2	p12	r2	879	9337	8808	1626	755	1327	24077	20587	21179	22398	24621	24146	0.04	0.45	0.42	0.07	0.03	0.05												
PIK3R1 v1	Experiment2	p12	r3	1630	9852	7890	2050	1611	2084	24369	21315	21004	22912	23869	24116	0.07	0.46	0.38	0.09	0.07	0.09												
PIK3R2	Experiment1	p7	r1	1191	4277	4578	4544	3311	4801	19410	20963	18593	19631	18565	20424	0.06	0.20	0.25	0.23	0.18	0.24	0.06	0.23	0.26	0.22	0.20	0.22	0.01	0.03	0.01	0.02	0.02	0.01
PIK3R2	Experiment1	p7	r2	1033	5185	5265	4248	4619	4659	21137	19598	19980	18857	20812	21245	0.05	0.26	0.26	0.23	0.22	0.22												
PIK3R2	Experiment1	p7	r3	1308	4929	5113	3759	4171	4481	22541	20898	19727	19053	20608	20657	0.06	0.24	0.26	0.20	0.20	0.22												
PIK3R2	Experiment2	p12	r1	1060	4948	5695	1885	1169	1367	22915	19627	21610	22202	22171	21326	0.05	0.25	0.26	0.08	0.05	0.06	0.05	0.28	0.28	0.09	0.06	0.08	0.01	0.02	0.07	0.01	0.01	0.01
PIK3R2	Experiment2	p12	r2	1368	6427	7281	2262	1264	1698	24958	22055	20577	22373	23727	22991	0.05	0.29	0.35	0.10	0.05	0.07												
PIK3R2	Experiment2	p12	r3	933	5395	4047	1935	1454	1852	20876	19083	18765	20519	21527	21140	0.04	0.28	0.22	0.09	0.07	0.09												
PTEN	Experiment2	p9	r1	1826	12401	10749	2570	1668	2774	29082	25531	24817	26938	27083	26539	0.06	0.49	0.43	0.10	0.06	0.10	0.07	0.54	0.44	0.10	0.07	0.11	0.00	0.06	0.09	0.01	0.01	0.01
PTEN	Experiment2	p9	r2	1994	15240	13246	3007	2120	3352	29605	25605	24895	27925	28585	26300	0.07	0.60	0.53	0.11	0.07	0.13												
PTEN	Experiment2	p9	r3	2009	13851	9189	2936	1803	3044	29714	25128	25787	28998	29013	29620	0.07	0.55	0.36	0.10	0.06	0.10												
PTPRA v1	Experiment2	p13	r1	1895	9590	11270	4068	2888	3493	23880	22030	25749	26672	26537	26211	0.08	0.44	0.44	0.15	0.11	0.13	0.08	0.47	0.44	0.14	0.10	0.14	0.01	0.04	0.04	0.02	0.04	0.03
PTPRA v1	Experiment2	p13	r2	1938	12061	11417	3081	1703	2676	26176	23828	23633	25857	25706	24119	0.07	0.51	0.48	0.12	0.07	0.11												
PTPRA v1	Experiment2	p13	r3	2165	11117	9427	3700	3726	4532	24552	23882	23388	26006	27295	27778	0.09	0.47	0.40	0.14	0.14	0.16												
PTPRE v2	Experiment2	p13	r1	3242	19101	17775	13996	7210	10825	27712	26613	26633	28988	29938	27392	0.12	0.72	0.67	0.48	0.24	0.40	0.12	0.84	0.83	0.53	0.28	0.47	0.01	0.16	0.31	0.09	0.06	0.08
PTPRE v2	Experiment2	p13	r2	3910	27191	30719	18088	10483	15568	31204	26775	25862	28748	29194	27830	0.13	1.02	1.19	0.63	0.36	0.56												
PTPRE v2	Experiment2	p13	r3	4156	22151	17764	13054	8113	13343	32288	28533	27642	27590	31960	29582	0.13	0.78	0.64	0.47	0.25	0.45												
PTPRO v4	Experiment2	p13	r1	3672	15778	18741	10533	6728	9185	26146	25532	26556	29505	30061	28722	0.14	0.62	0.71	0.36	0.22	0.32	0.12	0.70	0.68	0.31	0.21	0.34	0.04	0.07	0.06	0.07	0.06	0.06
PTPRO v4	Experiment2	p13	r2	2140	17396	15649	6625	4328	8086	27521	24451	25918	29123	30265	28079	0.08	0.71	0.60	0.23	0.14	0.29												
PTPRO v4	Experiment2	p13	r3	4228	20071	17422	9881	8074	11672	28893	26391	24257	27928	30034	28952	0.15	0.76	0.72	0.35	0.27	0.40												
PTPRR v2	Experiment2	p13	r1	4278	19731	20478	15432	9576	12706	26615	22943	24669	27819	30357	28713	0.16	0.86	0.83	0.55	0.32	0.44	0.13	0.88	0.81	0.48	0.29	0.42	0.05	0.05	0.05	0.09	0.04	0.02
PTPRR v2	Experiment2	p13	r2	2099	21035	19013	10686	6740	10487	28129	22451	22282	27725	27650	26256	0.07	0.94	0.85	0.39	0.24	0.40												
PTPRR v2	Experiment2	p13	r3	4449	20636	17899	13420	8893	12699	29408	24594	23610	27638	27925	29473	0.15	0.84	0.76	0.49	0.32	0.43												
RASA1 v1	Experiment2	p9	r1	2231	13081	13654	6828	5704	7377	26406	22974	24811	26322	28735	25111	0.08	0.57	0.55	0.26	0.20	0.29	0.08	0.57	0.57	0.26	0.19	0.30	0.02	0.02	0.06	0.04	0.01	0.02
RASA1 v1	Experiment2	p9	r2	1534	14091	14852	8011	5391	8483	28386	23735	23402	27408	27489	26479	0.05	0.59	0.63	0.29	0.20	0.32												
RASA1 v1	Experiment2	p9	r3	2881	14111	12416	6004	5465	7502	28637	25475	23981	27232	29345	27556	0.10	0.55	0.52	0.22	0.19	0.27												
RASA1 v1	Experiment4	p3	r1	1874	7081	6748	5882	4081	5042	12693	11470	11782	11864	11679	11090	0.15	0.62	0.57	0.50	0.35	0.45	0.13	0.67	0.58	0.49	0.31	0.42	0.02	0.05	0.04	0.01	0.04	0.03
RASA1 v1	Experiment4	p3	r2	1291	7272	6394	5369	3363	4342	11666	10244	10191	11096	11306	11082	0.11	0.71	0.63	0.48	0.30	0.39												
RASA1 v1	Experiment4	p3	r3	1754	7485	5820	4344	3083	4626	12709	11146	10660	8961	11079	10661	0.14	0.67	0.55	0.48	0.28	0.42												
RPS6KA1 v1	Experiment1	p1	r1	1945	11740	15382	12340	11525	10881	20704	19490	19067	17861	17342	17316	0.09	0.60	0.81	0.69	0.66	0.63	0.10	0.57	0.73	0.60	0.63	0.64	0.02	0.03	0.07	0.10	0.05	0.04
RPS6KA1 v1	Experiment1	p1	r2	1759	9134	11576	10895	9595	10373	20543	17030	17132	17625	16865	17046	0.09	0.54	0.68	0.62	0.57	0.61												
RPS6KA1 v1	Experiment1	p1	r3	2603	10796	12622	8822	12116	12582	21626	19274	18016	17668	18467	18424	0.12	0.56	0.70	0.50	0.66	0.68												
RPS6KA1 v1	Experiment2	p2	r1	1957	14993	15628	10359	6144	9626	24022	21366	20602	21934	22549	23941	0.08	0.70	0.76	0.47	0.27	0.40	0.10	0.80	0.78	0.58	0.32	0.43	0.01	0.17	0.17	0.15	0.08	0.06
RPS6KA1 v1	Experiment2	p2	r2	2697	21161	19845	17325	10112	11330	25098	21152	20799	23074	24258	22831	0.11	1.00	0.95	0.75	0.42	0.50												
RPS6KA1 v1	Experiment2	p2	r3	2256	14823	11757	10110	5736	8661	22837	21196	19128	20050	21321	21868	0.10	0.70	0.61	0.50	0.27	0.40												
RPS6KA2 v1	Experiment1	p1	r1	1490	9007	9429	8010	7931	8101	19058	16728	17308	16649	17257	15720	0.08	0.54	0.54	0.48	0.46	0.52	0.08	0.49	0.52	0.50	0.45	0.52	0.01	0.06	0.03	0.02	0.02	0.05
RPS6KA2 v1	Experiment1	p1	r2	1432	7296	8342	8058	7291	7192	18014	17109	17199	16383	15624	15314	0.08	0.43	0.49	0.49	0.47	0.47												
RPS6KA2 v1	Experiment1	p1	r3	1766	8944	8290	8630	7424	9687	19260	17628	16050	16821	17526	17183	0.09	0.51	0.52	0.51	0.42	0.56												
RPS6KA2 v1	Experiment2	p2	r1	1516	8455	10141	7244	4383	4997	24639	20435	22262	23562	23155	23231	0.06	0.41	0.46	0.31	0.19	0.22	0.05	0.48	0.48	0.34	0.19	0.23	0.01	0.12	0.13	0.08	0.04	0.03
RPS6KA2 v1	Experiment2	p2	r2	994	11838	13682	10132	5619	5569	23544	19048	22053	23521	24258	20649	0.04	0.61	0.62	0.43	0.23	0.27												
RPS6KA2 v1	Experiment2	p2	r3	1470	8957	7968	6136	3427	4660	24297	22238	21334	21218	22375	22600	0.06	0.40	0.37	0.29	0.15	0.21												
RPS6KA2 v1	Experiment4	p2	r1	1256	4548	3956	4572	2699	3285	10418	10298	10527	10069	10184	9995	0.12	0.44	0.38	0.45	0.27	0.33	0.10	0.41	0.37	0.42	0.23	0.31	0.03	0.05	0.01	0.03	0.03	0.04
RPS6KA2 v1	Experiment4	p2	r2	773	3514	3849	3944	2086	2469	11186	9901	10124	9839	10455	9383	0.07	0.35	0.38	0.40	0.20	0.26												
RPS6KA2 v1	Experiment4	p2	r3	1293	4810	3538	3784	2517	3435	11209	10815	10029	9644	10792	9710	0.12	0.44	0.35	0.39	0.23	0.35												
SASH1	Experiment1	p4	r1	1321	7332	8850	7301	6465	6708	15523	11450	11539	12455	12971	17052	0.09	0.64	0.77	0.59	0.50	0.39	0.09	0.69	0.73	0.60	0.54	0.44	0.00	0.09	0.04	0.02	0.05	0.05
SASH1	Experiment1	p4	r2	1564	11005	9485	9293	9433	8280	18669	13874	13853	14834	15795	16809	0.08	0.79	0.68	0.63	0.60	0.49												
SASH1	Experiment1	p4	r3	1429	7916	7782	7147	7164	7199	16217	12486	10553	11981	13342	16179	0.09	0.63	0.74	0.60	0.54	0.44												
SASH1	Experiment2	p3	r1	905	8989	8478	2072	1012	1474	20739	15992	17676	19964	19787	20552	0.04	0.56	0.48	0.10	0.05	0.07	0.05	0.59	0.50	0.10	0.05	0.08	0.01	0.05	0.09	0.00	0.00	0.01
SASH1	Experiment2	p3	r2	1130	10391	10970	2279	1132	1742	21098	15950	18476	21562	21206	21192	0.05	0.65	0.59	0.11	0.05	0.08												
SASH1	Experiment2	p3	r3	891	9522	7099	1996	1053	1666	20701	16994	16815	19870	19822	20239	0.04	0.56	0.42	0.10	0.05	0.08												
SASH1	Experiment4	p3	r1	526	3668	3581	2507	1014	2608	9640	7349	8948	8689	9369	8958	0.05	0.50	0.40	0.29	0.11	0.29	0.07	0.52	0.44	0.27	0.13	0.27	0.02	0.05	0.06	0.03	0.02	0.02
SASH1	Experiment4	p3	r2	684	3762	4061	2489	1124	2371	10028	7638	8040	8743	8894	8594	0.07	0.49	0.51	0.28	0.13	0.28												
SASH1	Experiment4	p3	r3	889	4351	2935	1888	1306	2063	8902	7493	7100	7843	8899	8094	0.10	0.58	0.41	0.24	0.15	0.25												
SH3BP1	Experiment2	p3	r1	1207	8818	9202	6625	4133	5168	23814	19116	21197	22167	22878	22208	0.05	0.46	0.43	0.30	0.18	0.23	0.05	0.49	0.46	0.31	0.18	0.26	0.00	0.05	0.08	0.02	0.01	0.05
SH3BP1	Experiment2	p3	r2	1059	10909	12027	7705	4445	6497	23870	20048	21820	23027	23144	20417	0.04	0.54	0.55	0.33	0.19	0.32												
SH3BP1	Experiment2	p3	r3	1239	9980	8034	7037	3912	5330	23480	21860	20370	23055	23787	23144	0.05	0.46	0.39	0.31	0.16	0.23												
SH3KBP1 v1	Experiment1	p5	r1	920	5993	6649	4939	4452	4958	16213	13360	12715	14219	13839	13801	0.06	0.45	0.52	0.35	0.32	0.36	0.07	0.48	0.60	0.45	0.41	0.42	0.01	0.04	0.11	0.12	0.11	0.08
SH3KBP1 v1	Experiment1	p5	r2	1457	7867	10474	8732	7948	8226	18058	15098	14504	14906	14822	16328	0.08	0.52	0.72	0.59	0.54	0.50												
SH3KBP1 v1	Experiment1	p5	r3	1258	6858	7781	6818	6545	7034	16473	14892	14188	15969	18265	17843	0.08	0.46	0.55	0.43	0.36	0.39												
SH3KBP1 v1	Experiment2	p2	r1	1516	12556	12419	7464	5438	7021	24876	21472	23813	23966	26648	24862	0.06	0.58	0.52	0.31	0.20	0.28	0.06	0.60	0.54	0.34	0.22	0.30	0.00	0.09	0.10	0.06	0.02	0.04
SH3KBP1 v1	Experiment2	p2	r2	1109	13031	12748	9174	5305	7801	21322	18848	19625	22052	21717	23085	0.05	0.69	0.65	0.42	0.24	0.34												
SH3KBP1 v1	Experiment2	p2	r3	1379	10838	9575	6790	5048	6202	25388	20858	20845	22828	24541	22561	0.05	0.52	0.46	0.30	0.21	0.27												
SNF1LK	Experiment1	p4	r1	2110	11018	14076	13230	11177	12425	18673	15544	16056	17662	18002	18086	0.11	0.71	0.88	0.75	0.62	0.69	0.11	0.77	0.87	0.76	0.68	0.70	0.02	0.06	0.03	0.05	0.06	0.07
SNF1LK	Experiment1	p4	r2	2066	13479	14020	14387	12228	12132	22073	16434	16842	17665	18240	19063	0.09	0.82	0.83	0.81	0.67	0.64												
SNF1LK	Experiment1	p4	r3	2745	14060	14631	11929	13174	14321	21229	18332	16309	16662	17824	18390	0.13	0.77	0.90	0.72	0.74	0.78												
SNF1LK	Experiment2	p1	r1	1114	12558	11119	7886	4219	5423	17934	16147	16168	15798	15811	14307	0.06	0.78	0.69	0.50	0.27	0.38	0.06	0.84	0.78	0.57	0.32	0.41	0.01	0.15	0.22	0.15	0.09	0.06
SNF1LK	Experiment2	p1	r2	914	13848	14134	10490	6000	6962	16842	13709	13711	14235	14397	14719	0.05	1.01	1.03	0.74	0.42	0.47												
SNF1LK	Experiment2	p1	r3	1326	10642	8025	6338	3807	5320	17392	14413	12962	13810	14448	14484	0.08	0.74	0.62	0.46	0.26	0.37												
STAT5A	Experiment4	p1	r1	1611	6909	6229	5313	3311	4756	11094	9459	10198	10151	10336	10194	0.15	0.73	0.61	0.52	0.32	0.47	0.13	0.69	0.59	0.53	0.28	0.43	0.04	0.06	0.04	0.04	0.04	0.04
STAT5A	Experiment4	p1	r2	889	5473	5568	4921	2799	4398	10266	8838	9230	10076	11462	10764	0.09	0.62	0.60	0.49	0.24	0.41												
STAT5A	Experiment4	p1	r3	1621	7451	5555	5123	2708	3932	10778	10307	10233	9095	9822	9743	0.15	0.72	0.54	0.56	0.28	0.40												
WBP2	Experiment1	p5	r1	1081	4495	5543	4163	3834	3785	14652	12149	12890	12579	13393	13609	0.07	0.37	0.43	0.33	0.29	0.28	0.08	0.45	0.50	0.38	0.32	0.31	0.01	0.07	0.09	0.08	0.06	0.05
WBP2	Experiment1	p5	r2	1136	6497	7942	6120	5165	5594	15385	12835	13158	12915	13291	15154	0.07	0.51	0.60	0.47	0.39	0.37												
WBP2	Experiment1	p5	r3	1398	6314	6534	5675	4746	5126	16511	13510	14133	16287	16471	17415	0.08	0.47	0.46	0.35	0.29	0.29												
WBP2	Experiment2	p3	r1	499	8561	7956	5248	2697	4540	21533	18606	20703	21162	21302	22222	0.02	0.46	0.38	0.25	0.13	0.20	0.03	0.50	0.43	0.27	0.14	0.21	0.01	0.09	0.07	0.03	0.02	0.02
WBP2	Experiment2	p3	r2	588	12111	11519	6720	3665	5218	23377	20106	22176	22288	23563	22372	0.03	0.60	0.52	0.30	0.16	0.23												
WBP2	Experiment2	p3	r3	960	8980	7794	5496	3287	4401	23715	20003	19520	21261	21888	23754	0.04	0.45	0.40	0.26	0.15	0.19												
WBP2	Experiment4	p2	r1	929	4155	4092	4194	2224	2992	11483	9281	10777	10628	10076	8908	0.08	0.45	0.38	0.39	0.22	0.34	0.08	0.48	0.41	0.37	0.24	0.33	0.01	0.03	0.05	0.02	0.02	0.01
WBP2	Experiment4	p2	r2	762	4594	4399	3571	2614	3161	10850	9008	9451	10046	10369	9783	0.07	0.51	0.47	0.36	0.25	0.32												
WBP2	Experiment4	p2	r3	868	4610	3928	3766	2511	3257	11620	9848	10174	10083	10697	9441	0.07	0.47	0.39	0.37	0.23	0.34												
